# Supplementary material for: Decreasing human body temperature in the United States since the Industrial Revolution
Source: eLife. 2020 Jan 7;9:e49555. doi: 10.7554/eLife.49555 (PMC6946399; doi:10.7554/eLife.49555)
Supplement: Source code 2. [file elife-49555-code2.zip › Temperature paper analysis markdown file PDF 20191104.pdf]

Temperature paper analysis

```
library(easyGgplot2)
#ggplot2.multiplot(..., plotlist=NULL, cols=2)
library(easyGgplot2)
library(lme4)
library(merTools)
combinedData <- read.csv("~/Desktop/Temperature study/combined_data.csv")

combinedData$time_HR_imputed <- combinedData$time_HR
combinedData$time_HR_imputed[is.na(combinedData$time_HR_imputed)] <- 12

dataVeterans  <- read.csv('~/Desktop/Temperature study/veterans_processed.csv')
dataNHANES    <- read.csv('~/Desktop/Temperature study/NHANES_processed.csv')
ST_data       <- read.csv('~/Desktop/Temperature study/STRIDE_processed.csv')

dataVeterans$race <- factor(as.character(dataVeterans$race),levels = c("white", "b
lack"))
combinedData$race [combinedData$race=="asian"]<- "other"
combinedData$race <- factor(combinedData$race,levels = c("white", "black","other"
))
dataNHANES$race[dataNHANES$race == "unknown"] <- NA
dataNHANES$race <- factor(dataNHANES$race,levels = c("white", "black","other" ))
ST_data$race [ST_data$race=="Asian"]<- "Other"
ST_data$race <- factor(ST_data$race,levels = c("White", "Black","Other"))
ST_data$exam_month<-factor (ST_data$exam_month)

dataVeterans$state_type <- factor(dataVeterans$state_type, levels = c("very cold",
"cold","moderately_cold","moderate","warm", "hot"))

dataNHANES$exammonth <-as.factor(dataNHANES$exammonth)
dataVeterans$exammonth <-as.factor(dataVeterans$exammonth)


state_temps <- read.csv("~/Desktop/Temperature study/state_temps.csv")
state_codes <- read.csv("~/Desktop/Temperature study/state_codes.csv")
state_codes$Code <- factor(as.character(state_codes$Code), levels=levels(dataVeter
ans$state))
state_temps$state_code <- state_codes$Code[match(as.character(state_temps$state),a
s.character(state_codes$State))]

dataVeterans$ambient_temp <- (state_temps$value[match(paste(dataVeterans$state,(da
taVeterans$examyear * 100 + as.numeric(dataVeterans$exammonth))), paste(state_temp
s$state_code,state_temps$date))] - 32) / 1.8

ST_data$state <- "CA"

ST_data$ambient_temp <- (state_temps$value[match(paste(ST_data$state,(ST_data$exam
_year * 100 + as.numeric(ST_data$exam_month))), paste(state_temps$state_code,state
_temps$date))] - 32) / 1.8
```

```
ggplot(combinedData, aes(x=temp_C)) + geom_histogram(binwidth = 0.1, center=37, ae
s(y = ..ncount..)) + theme(legend.position="top", axis.text=element_text(size=12),
axis.title=element_text(size=14,face="bold"), legend.text=element_text(size=12), l
egend.title=element_text(size=14)) + facet_grid(period ~ .) + labs( x="Temperature
,°C", y="Share of Observations", colour="", fill="") + scale_x_continuous(breaks=se
q(35,39,0.5), minor_breaks=seq(35,39,0.1))
```

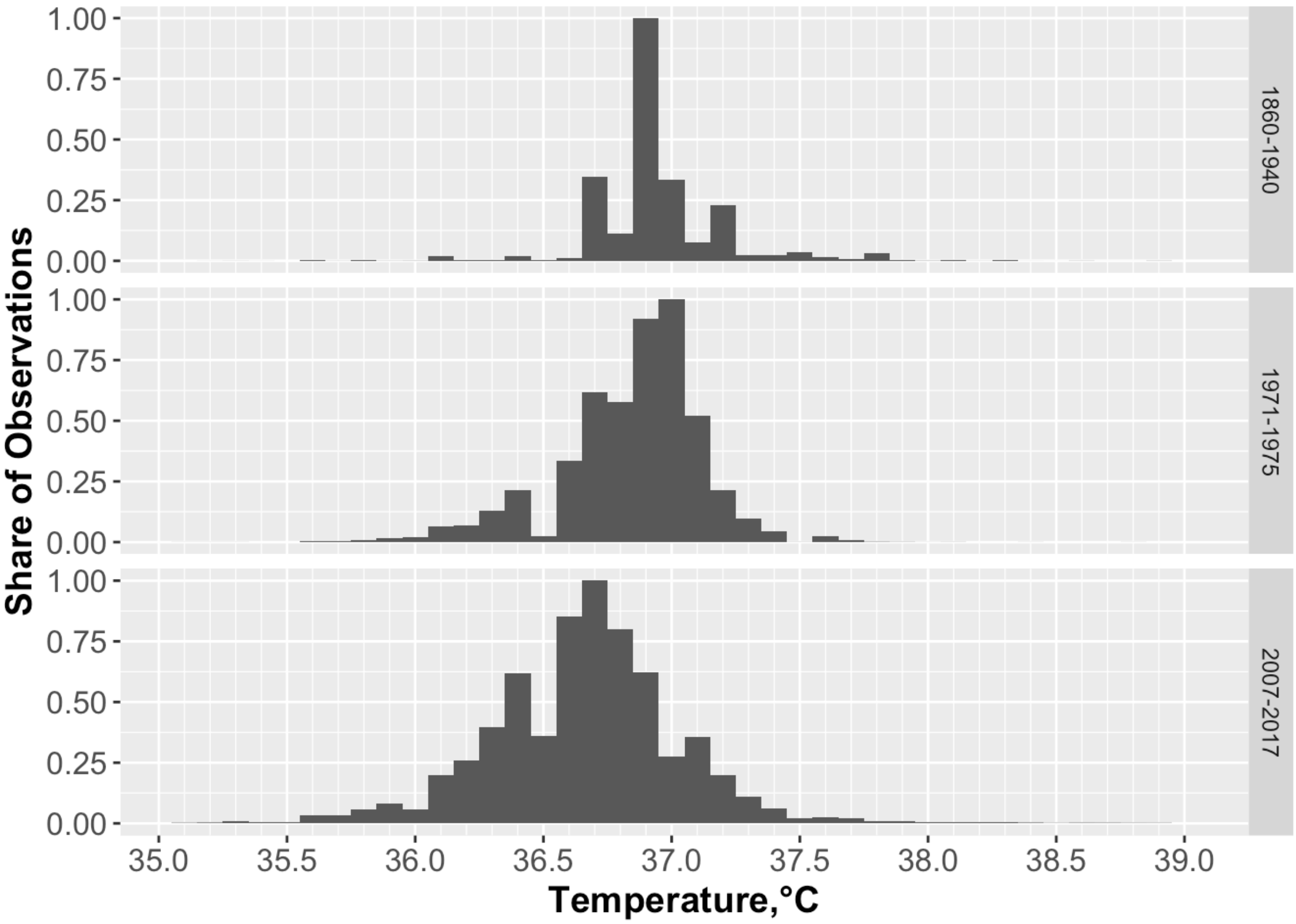


```
ggplot(combinedData, aes(x=temp)) + geom_histogram(binwidth = 0.1, center=98.6, ae
s(y = ..ncount..)) + theme(legend.position="top", axis.text=element_text(size=12),
axis.title=element_text(size=14,face="bold"), legend.text=element_text(size=12), l
egend.title=element_text(size=14)) + facet_grid(period ~ .) + labs( x="Temperature
,°F", y="Share of Observations", colour="", fill="") + scale_x_continuous(breaks=se
q(95,103,1), minor_breaks=seq(95,103,0.2))
```

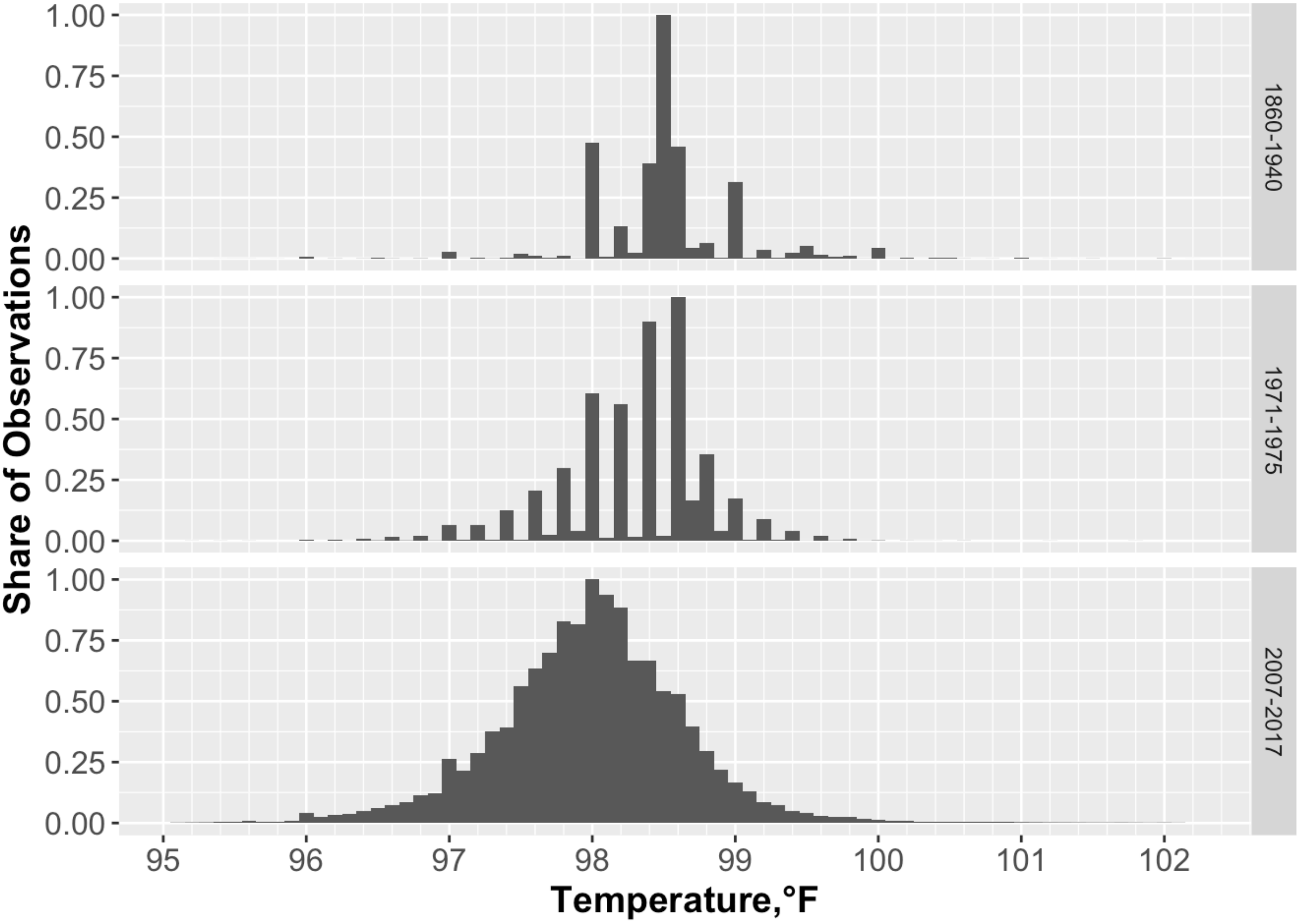

This is our analysis step by step. We first present a plot with the data from all the cohorts for white men and women separately. Body temperature measurements by age as observed in three different time periods/cohorts (smoothed unadjusted data)

```
gg_color_hue <- function(n) {
  hues = seq(15, 375, length = n + 1)
  hcl(h = hues, l = 65, c = 100)[1:n]
}
#Names("Black", "Orange", "Cyan", "Lt.Green", "Dk.Gray", "Red", "Blue", "Green", "
Brown", "Purple", "Lt.Gray", "Yellow", "Pink"))
#c("#000000", "#FF9233", "#29D0D0", "#81C57A", "#575757", "#AD2323", "#2A4BD7", "#
1D6914", "#814A19", "#8126C0", "#A0A0A0", "#FFEE33", "#FFCDF3")

plot_colors <- c("#FF9233", "#29D0D0", "#81C57A", "#575757", "#8126C0", "#29D0D0"
,"#575757", "#AD2323", "#2A4BD7", "#1D6914", "#814A19", "#8126C0", "#A0A0A0", "#FF
EE33", "#FFCDF3") # gg_color_hue(3)

combinedData$plot_sex[combinedData$sex=="male"] <- "men"
 combinedData$plot_sex[combinedData$sex=="female"] <- "women"
 combinedData$plot_sex <- factor(combinedData$plot_sex,levels=c("men","women"))

 Combined <- ggplot(data = combinedData[ combinedData$age  >30  &  combinedData$ag
e
 <80 & combinedData$race %in% c("white","black"),],aes(age, temp_C, group=period,c
olour=period)) + geom_smooth() + scale_colour_manual(values = plot_colors[c(2,3,1
)
 ]) + ylim(36,37.5) + facet_grid(plot_sex ~ race)

 Combined + theme(legend.position="bottom", axis.text=element_text(size=14),
         axis.title=element_text(size=14,face="bold"), legend.text=element_text(si
ze=12), legend.title=element_text(size=14)) + labs(x= "Age, years", y = "Temperatu
r
e,°C in white men(smoothed unadjusted data)", colour = "Time period of the measurem
ement")
```

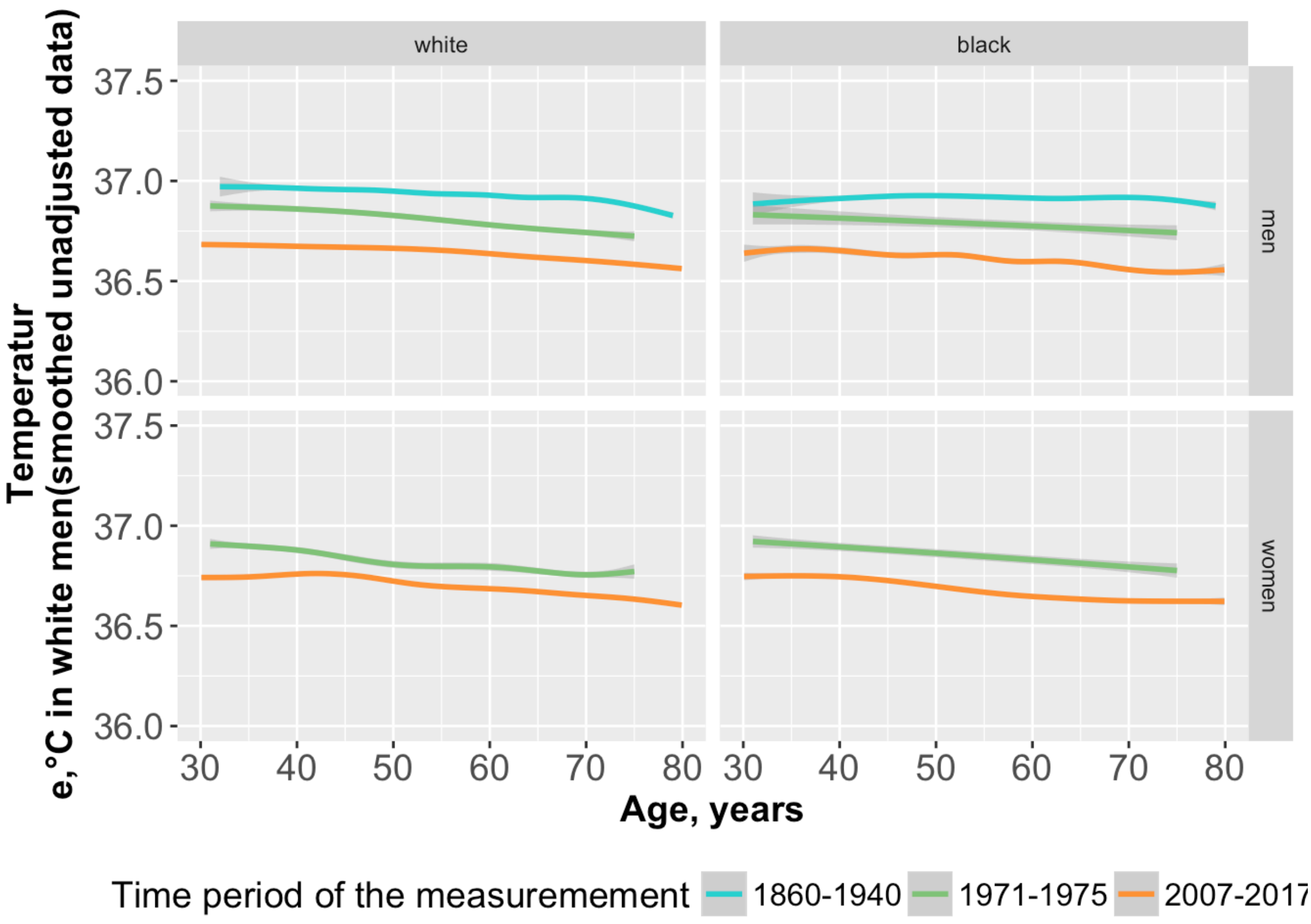

Figure 1 A: Body temperature measurements by age as observed in three different time periods (cohorts)

Then we run separate regressions for each cohort and present it in a table in results : lmVets- regression from Union Army veterans cohort from 1860-1940, NHANES data from 1971-1975 and STRIDE data from 2000-2017. STRIDE and a part of NHANES also includes time of the day (Time_HR). We also do predictions for men and women at age 30, body weight 70KG and height 170 CM, to compare the values across different cohorts.

```
lmVeterans <-lm(temp_C~ age + weightKG + heightCM + race, data= dataVeterans)
summary(lmVeterans)
```

```
##
## Call:
## lm(formula = temp_C ~ age + weightKG + heightCM + race, data = dataVeterans)
##
## Residuals:
##      Min       1Q   Median       3Q      Max
## -1.83518 -0.08302 -0.00718  0.06855  2.05607
##
## Coefficients:
##               Estimate Std. Error  t value Pr(>|t|)
## (Intercept)  3.714e+01  2.457e-02 1511.148   <2e-16 ***
## age         -2.987e-03  1.028e-04  -29.063   <2e-16 ***
## weightKG    -2.232e-04  9.283e-05   -2.405   0.0162 *
## heightCM     1.670e-05  1.444e-04    0.116   0.9079
## raceblack   -2.095e-02  2.115e-03   -9.907   <2e-16 ***
## ---
## Signif. codes:  0 '***' 0.001 '**' 0.01 '*' 0.05 '.' 0.1 ' ' 1
##
## Residual standard error: 0.2616 on 83895 degrees of freedom
## Multiple R-squared:  0.0116, Adjusted R-squared:  0.01156
## F-statistic: 246.2 on 4 and 83895 DF,  p-value: < 2.2e-16
```

```
lmVeterans_predict=data.frame(age=30, weightKG=70, heightCM = 170, race="white")

round(predict (lmVeterans, lmVeterans_predict, interval="confidence"),2)
```

```
##     fit   lwr   upr
## 1 37.03 37.03 37.04
```

NHANES analysis controlling for race and time of the day

In NHANES we included weights from the sample to try and get adjusted values, because we know that NHANES for example oversampled women in fertile age. But the coefficients in the weighted model were not very different from the initial model.

```
lmNHANES_W_races <- lm(temp_C ~ age + weight_KG + height_CM + race+ time_HR, data=
 dataNHANES[dataNHANES$sex == "male" , ], weights=dataNHANES$sample_weights[dataNH
ANES$sex=="male"])
 summary(lmNHANES_W_races)
```

```
##
## Call:
## lm(formula = temp_C ~ age + weight_KG + height_CM + race + time_HR,
##     data = dataNHANES[dataNHANES$sex == "male", ], weights = dataNHANES$sample_
weights[dataNHANES$sex ==
##         "male"])
##
## Weighted Residuals:
##      Min       1Q   Median       3Q      Max
## -181.125   -0.139    0.000    7.172  143.973
##
## Coefficients:
##               Estimate Std. Error t value Pr(>|t|)
## (Intercept) 36.7386941  0.1814071 202.521  < 2e-16 ***
## age         -0.0025635  0.0005415  -4.734 2.38e-06 ***
## weight_KG    0.0009050  0.0005124   1.766   0.0776 .
## height_CM   -0.0002749  0.0010588  -0.260   0.7952
## raceblack   -0.0099886  0.0234635  -0.426   0.6704
## raceother    0.1178001  0.0663093   1.777   0.0758 .
## time_HR      0.0124873  0.0016484   7.575 5.75e-14 ***
## ---
## Signif. codes:  0 '***' 0.001 '**' 0.01 '*' 0.05 '.' 0.1 ' ' 1
##
## Residual standard error: 31.15 on 1759 degrees of freedom
##   (2995 observations deleted due to missingness)
## Multiple R-squared:  0.05296,    Adjusted R-squared:  0.04973
## F-statistic:  16.4 on 6 and 1759 DF,  p-value: < 2.2e-16
```

```
NHANES_predict=data.frame (age=30, weight_KG=70, height_CM = 170, race="white", se
x ="male", time_HR=12)
round(predict(lmNHANES_W_races, NHANES_predict, interval="confidence"),2)
```

```
##     fit  lwr   upr
## 1 36.83 36.8 36.86
```

```
lmNHANES_W_races_F <- lm(temp_C ~ age + weight_KG + height_CM + race+ time_HR, dat
a=dataNHANES[dataNHANES$sex == "female" , ], weights=dataNHANES$sample_weights[dat
aNHANES$sex=="female"])
summary(lmNHANES_W_races_F)
```

```
##
## Call:
## lm(formula = temp_C ~ age + weight_KG + height_CM + race + time_HR,
##     data = dataNHANES[dataNHANES$sex == "female", ], weights = dataNHANES$sampl
e_weights[dataNHANES$sex ==
##         "female"])
##
## Weighted Residuals:
##      Min       1Q   Median       3Q      Max
## -215.060    0.000    0.000    6.141  133.405
##
## Coefficients:
##               Estimate Std. Error t value Pr(>|t|)
## (Intercept)  3.680e+01  1.844e-01 199.492  < 2e-16 ***
## age         -3.076e-03  5.587e-04  -5.505 4.17e-08 ***
## weight_KG    5.576e-04  4.451e-04   1.253    0.210
## height_CM   -1.991e-05  1.109e-03  -0.018    0.986
## raceblack    1.006e-02  2.283e-02   0.441    0.660
## raceother    5.422e-02  7.480e-02   0.725    0.469
## time_HR      9.954e-03  1.771e-03   5.621 2.17e-08 ***
## ---
## Signif. codes:  0 '***' 0.001 '**' 0.01 '*' 0.05 '.' 0.1 ' ' 1
##
## Residual standard error: 27.33 on 1940 degrees of freedom
##   (5774 observations deleted due to missingness)
## Multiple R-squared:  0.03325,    Adjusted R-squared:  0.03026
## F-statistic: 11.12 on 6 and 1940 DF,  p-value: 3.164e-12
```

```
NHANES_predict=data.frame (age=30, weight_KG=70, height_CM = 170, race="white", se
x ="female", time_HR=12)
 round(predict(lmNHANES_W_races_F, NHANES_predict, interval="confidence"),2)
```

```
##     fit   lwr   upr
## 1 36.86 36.83 36.89
```

STRIDE models including all races for men and women

```
lmSTRIDE_races <- lm(temp_C ~ age_years +  weight_KG + height_CM + race + time_HR
 ,data=ST_data[ST_data$GENDER == "Male" , ])
 summary(lmSTRIDE_races)
```

```
##
## Call:
## lm(formula = temp_C ~ age_years + weight_KG + height_CM + race +
##     time_HR, data = ST_data[ST_data$GENDER == "Male", ])
##
## Residuals:
##      Min       1Q   Median       3Q      Max
## -1.70429 -0.20296  0.00938  0.21149  2.45466
##
## Coefficients:
##               Estimate Std. Error  t value Pr(>|t|)
## (Intercept)  3.667e+01  2.070e-02 1771.454   <2e-16 ***
## age_years   -2.938e-03  5.314e-05  -55.297   <2e-16 ***
## weight_KG    1.141e-03  4.920e-05   23.187   <2e-16 ***
## height_CM   -1.531e-03  1.175e-04  -13.021   <2e-16 ***
## raceBlack   -6.045e-02  3.108e-03  -19.451   <2e-16 ***
## raceOther   -9.305e-04  1.885e-03   -0.494    0.622
## time_HR      2.310e-02  2.874e-04   80.370   <2e-16 ***
## ---
## Signif. codes:  0 '***' 0.001 '**' 0.01 '*' 0.05 '.' 0.1 ' ' 1
##
## Residual standard error: 0.3634 on 210795 degrees of freedom
##   (19414 observations deleted due to missingness)
## Multiple R-squared:  0.04922,    Adjusted R-squared:  0.0492
## F-statistic:  1819 on 6 and 210795 DF,  p-value: < 2.2e-16
```

```
 STRIDE_predict=data.frame(age_years=30, weight_KG=70, height_CM = 170, time_HR=12
,
race="White", GENDER = "Male")
round(predict(lmSTRIDE_races, STRIDE_predict, interval="confidence"),2)
```

```
##     fit   lwr   upr
## 1 36.68 36.68 36.68
```

```
lmSTRIDE_F_races <- lm(temp_C ~ age_years +  weight_KG + height_CM + race+time_HR
,data=ST_data[ST_data$GENDER == "Female" , ])
summary(lmSTRIDE_F_races)
```

```
##
## Call:
## lm(formula = temp_C ~ age_years + weight_KG + height_CM + race +
##     time_HR, data = ST_data[ST_data$GENDER == "Female", ])
##
## Residuals:
##     Min      1Q  Median      3Q     Max
## -1.7380 -0.2016  0.0016  0.2063  2.4019
##
## Coefficients:
##               Estimate Std. Error t value Pr(>|t|)
## (Intercept)  3.679e+01  1.610e-02 2285.16  < 2e-16 ***
## age_years   -3.677e-03  4.120e-05  -89.25  < 2e-16 ***
## weight_KG    6.986e-04  3.818e-05   18.30  < 2e-16 ***
## height_CM   -1.205e-03  9.589e-05  -12.56  < 2e-16 ***
## raceBlack   -5.226e-02  2.251e-03  -23.22  < 2e-16 ***
## raceOther   -7.512e-03  1.459e-03   -5.15 2.61e-07 ***
## time_HR      2.106e-02  2.346e-04   89.77  < 2e-16 ***
## ---
## Signif. codes:  0 '***' 0.001 '**' 0.01 '*' 0.05 '.' 0.1 ' ' 1
##
## Residual standard error: 0.3564 on 322415 degrees of freedom
##   (25584 observations deleted due to missingness)
## Multiple R-squared:  0.05082,    Adjusted R-squared:  0.0508
## F-statistic:  2877 on 6 and 322415 DF,  p-value: < 2.2e-16
```

```
STRIDE_predict=data.frame(age_years=30, weight_KG=70, height_CM = 170, time_HR=12,
 race="White", GENDER = "Female")
 round(predict(lmSTRIDE_F_races, STRIDE_predict, interval="confidence"),2)
```

```
##     fit   lwr   upr
## 1 36.77 36.77 36.77
```

Month effect analysis

```
library(arm)
lmerST_data_month <-lmer(temp_C~ age_years + weight_KG + height_CM + (1 | exam_mon
th), data= ST_data [ST_data$GENDER == "Male" & ST_data$race == "White",])
summary(lmerST_data_month)
```

```
## Linear mixed model fit by REML ['lmerMod']
## Formula: temp_C ~ age_years + weight_KG + height_CM + (1 | exam_month)
##    Data: ST_data[ST_data$GENDER == "Male" & ST_data$race == "White", ]
##
## REML criterion at convergence: 104143.1
##
## Scaled residuals:
##     Min      1Q  Median      3Q     Max
## -4.4738 -0.5580  0.0275  0.5811  6.4950
##
## Random effects:
##  Groups     Name        Variance  Std.Dev.
##  exam_month (Intercept) 0.0000414 0.006435
##  Residual               0.1327657 0.364370
## Number of obs: 127113, groups:  exam_month, 12
##
## Fixed effects:
##               Estimate Std. Error t value
## (Intercept)  3.703e+01  2.614e-02  1416.4
## age_years   -3.192e-03  6.886e-05   -46.4
## weight_KG    1.253e-03  6.282e-05    19.9
## height_CM   -1.958e-03  1.501e-04   -13.0
##
## Correlation of Fixed Effects:
##           (Intr) ag_yrs wgh_KG
## age_years -0.305
## weight_KG  0.192 -0.112
## height_CM -0.967  0.179 -0.386
```

```
lmerST_data_month_re <- data.frame(re=as.numeric(ranef(lmerST_data_month)$exam_mon
th[,1]), se=as.numeric(se.ranef(lmerST_data_month)$exam_month[,1]))
lmerST_data_month_re$low <- lmerST_data_month_re$re - 1.96 * lmerST_data_month_re$
se
lmerST_data_month_re$high <- lmerST_data_month_re$re + 1.96 * lmerST_data_month_re
$se
lmerST_data_month_re$month <- factor(c("January","February","March","April","May",
"June","July","August","September","October","November","December"),levels=c("Janu
ary","February","March","April","May","June","July","August","September","October"
,"November","December"))

lmerST_data_month_re$sex <- "men"
lmerST_data_month_re$race <- "white"
lmerST_data_month_re$period <- "STRIDE"

ggplot(data=lmerST_data_month_re,aes(x=month, y=re, color = "blue")) + geom_point(
) + geom_linerange(aes(ymin=low, ymax=high)) + labs(title="Month effect on tempera
ture in white men STRIDE", x="", y="Temperature Difference, C", colour="", fill=""
) + theme(legend.position="none", axis.text.x = element_text(angle = 30, hjust = 1
))
```

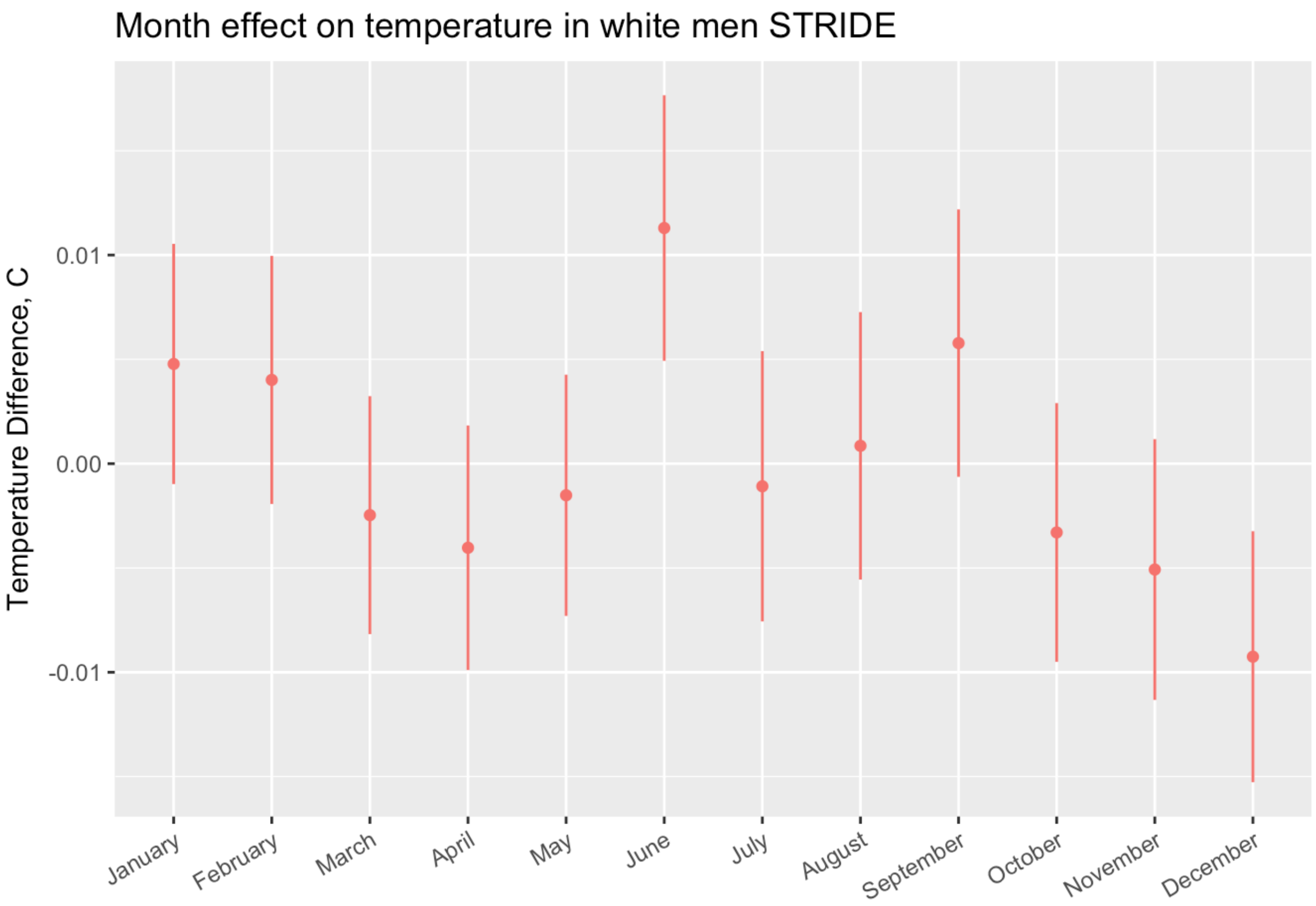


```
library(arm)
lmerST_data_month_F <-lmer(temp_C~ age_years + weight_KG + height_CM + (1 | exam_m
onth), data= ST_data [ST_data$GENDER == "Female" & ST_data$race == "White",])
summary(lmerST_data_month_F)
```

```
## Linear mixed model fit by REML ['lmerMod']
## Formula: temp_C ~ age_years + weight_KG + height_CM + (1 | exam_month)
##    Data: ST_data[ST_data$GENDER == "Female" & ST_data$race == "White",
##     ]
##
## REML criterion at convergence: 141063.7
##
## Scaled residuals:
##     Min      1Q  Median      3Q     Max
## -4.6635 -0.5733 -0.0018  0.5786  6.4769
##
## Random effects:
##  Groups     Name        Variance  Std.Dev.
##  exam_month (Intercept) 6.738e-05 0.008209
##  Residual               1.290e-01 0.359167
## Number of obs: 178468, groups:  exam_month, 12
##
## Fixed effects:
##               Estimate Std. Error t value
## (Intercept)  3.703e+01  2.103e-02  1760.9
## age_years   -3.727e-03  5.554e-05   -67.1
## weight_KG    7.915e-04  5.046e-05    15.7
## height_CM   -1.162e-03  1.263e-04    -9.2
##
## Correlation of Fixed Effects:
##           (Intr) ag_yrs wgh_KG
## age_years -0.356
## weight_KG  0.107 -0.095
## height_CM -0.969  0.233 -0.274
```

```
lmerST_data_month_F_re <- data.frame(re=as.numeric(ranef(lmerST_data_month_F)$exam
_month[,1]), se=as.numeric(se.ranef(lmerST_data_month_F)$exam_month[,1]))
lmerST_data_month_F_re$low <- lmerST_data_month_F_re$re - 1.96 * lmerST_data_month
_F_re$se
lmerST_data_month_F_re$high <- lmerST_data_month_F_re$re + 1.96 * lmerST_data_mont
h_F_re$se
lmerST_data_month_F_re$month <- factor(c("January","February","March","April","May
","June","July","August","September","October","November","December"),levels=c("Ja
nuary","February","March","April","May","June","July","August","September","Octobe
r","November","December"))

lmerST_data_month_F_re$sex <- "women"
lmerST_data_month_F_re$race <- "white"
lmerST_data_month_F_re$period <- "STRIDE"

ggplot(data=lmerST_data_month_F_re,aes(x=month, y=re, color = "red")) + geom_point
() + geom_linerange(aes(ymin=low, ymax=high)) + labs(title="Month effect on temper
ature in white women in STRIDE", x="", y="Temperature Difference, C", colour="", f
ill="") + theme(legend.position="none", axis.text.x = element_text(angle = 30, hju
st = 1))
```

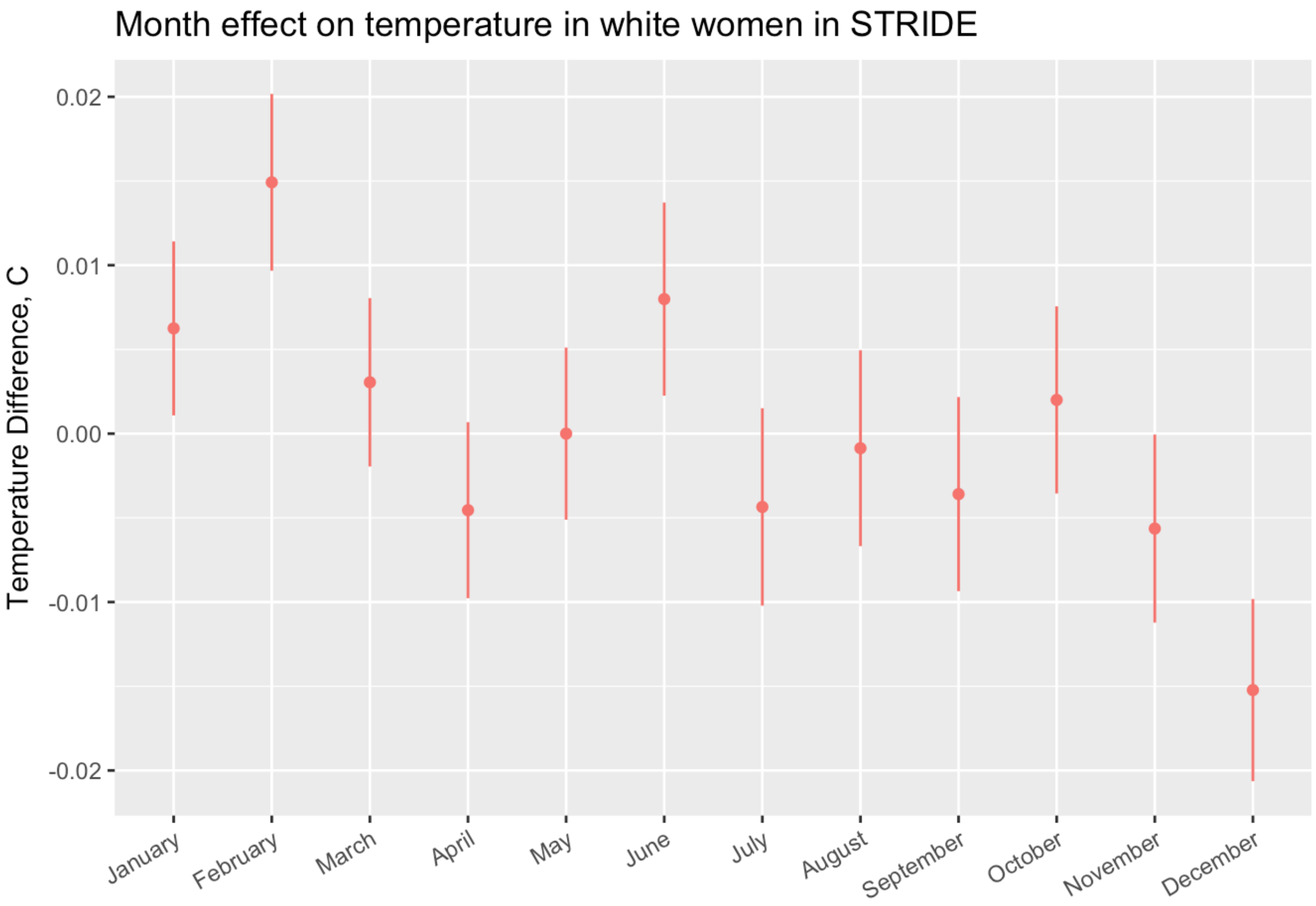

```
library(arm)
lmerVeterans_month <-lmer(temp_C~ age + weightKG + heightCM + (1 | exammonth), dat
a= dataVeterans [dataVeterans$race == "white",])
summary(lmerVeterans_month)
```

```
## Linear mixed model fit by REML ['lmerMod']
## Formula: temp_C ~ age + weightKG + heightCM + (1 | exammonth)
##    Data: dataVeterans[dataVeterans$race == "white", ]
##
## REML criterion at convergence: 15451.2
##
## Scaled residuals:
##     Min      1Q  Median      3Q     Max
## -6.7140 -0.3468 -0.0453  0.2834  7.4563
##
## Random effects:
##  Groups    Name        Variance  Std.Dev.
##  exammonth (Intercept) 0.0002193 0.01481
##  Residual              0.0747550 0.27341
## Number of obs: 62882, groups:  exammonth, 12
##
## Fixed effects:
##               Estimate Std. Error t value
## (Intercept)  3.717e+01  2.995e-02  1240.9
## age         -3.528e-03  1.223e-04   -28.8
## weightKG    -1.825e-04  1.093e-04    -1.7
## heightCM    -1.924e-05  1.730e-04    -0.1
##
## Correlation of Fixed Effects:
##          (Intr) age    wghtKG
## age      -0.326
## weightKG  0.102  0.021
## heightCM -0.930  0.089 -0.357
```

```
lmerVeterans_month_re <- data.frame(re=as.numeric(ranef(lmerVeterans_month)$exammo
nth[,1]), se=as.numeric(se.ranef(lmerVeterans_month)$exammonth[,1]))
lmerVeterans_month_re$low <- lmerVeterans_month_re$re - 1.96 * lmerVeterans_month_
re$se
lmerVeterans_month_re$high <- lmerVeterans_month_re$re + 1.96 * lmerVeterans_month
_re$se
lmerVeterans_month_re$month <- factor(c("January","February","March","April","May"
,"June","July","August","September","October","November","December"),levels=c("Jan
uary","February","March","April","May","June","July","August","September","October
","November","December"))

lmerVeterans_month_re$sex <- "men"
lmerVeterans_month_re$race <- "white"
lmerVeterans_month_re$period <- "UAVCW"
ggplot(data=lmerVeterans_month_re,aes(x=month, y=re, color = "red")) + geom_point(
) + geom_linerange(aes(ymin=low, ymax=high)) + labs(title="Month effect on tempera
ture in white men UAVCW", x="", y="Temperature Difference, C", colour="", fill="")
+ theme(legend.position="none", axis.text.x = element_text(angle = 30, hjust = 1))
```

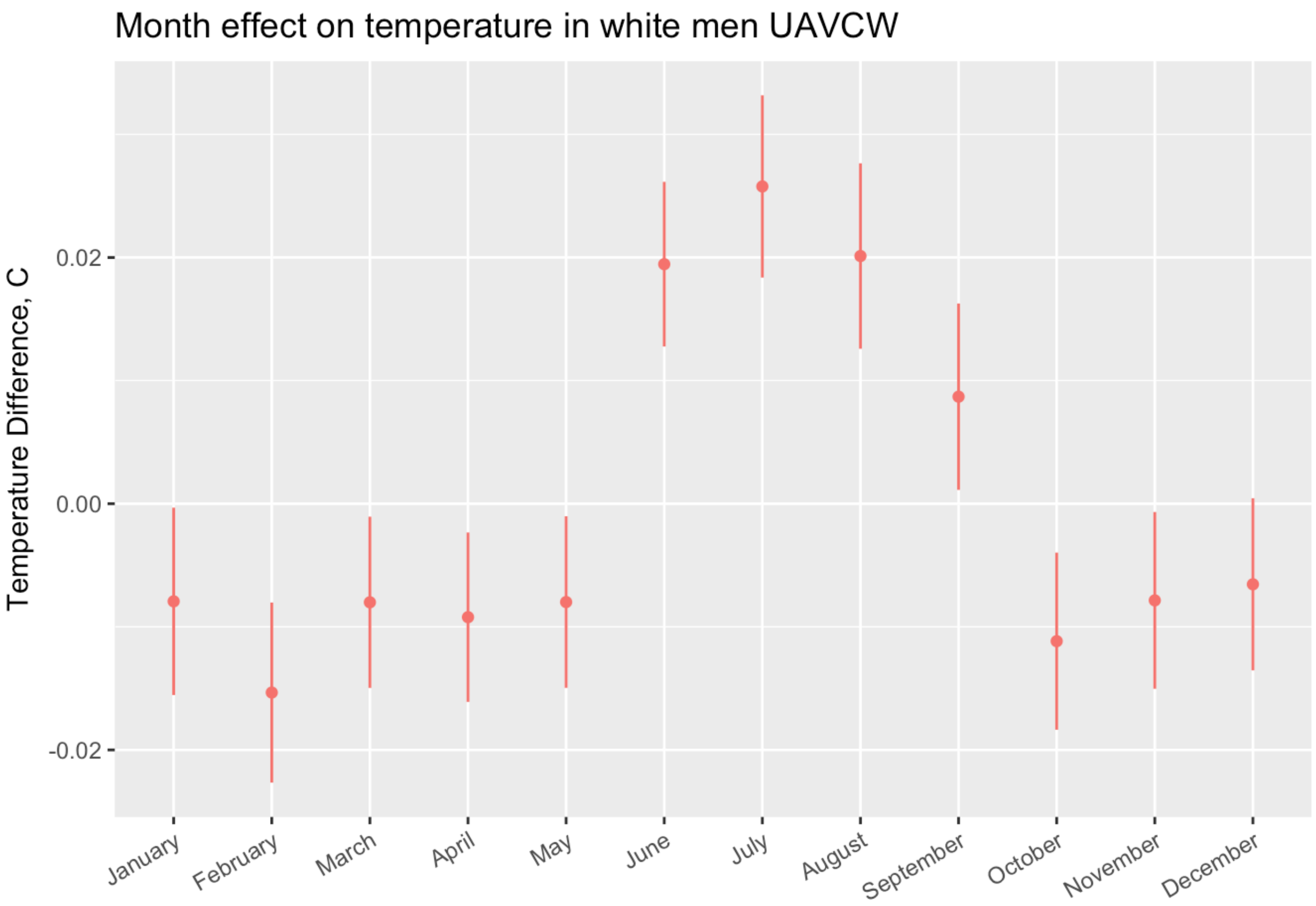

```
 lmerNHANES_month <-lmer(temp_C ~ age + weight_KG + height_CM + (1 | exammonth), d
ata=dataNHANES[dataNHANES$sex == "male" & dataNHANES$race == "white", ], weights=d
ataNHANES$sample_weights[dataNHANES$sex=="male" & dataNHANES$race == "white"])
summary(lmerNHANES_month)
```

```
## Linear mixed model fit by REML ['lmerMod']
## Formula: temp_C ~ age + weight_KG + height_CM + (1 | exammonth)
##    Data:
## dataNHANES[dataNHANES$sex == "male" & dataNHANES$race == "white",      ]
## Weights:
## dataNHANES$sample_weights[dataNHANES$sex == "male" & dataNHANES$race ==
##     "white"]
##
## REML criterion at convergence: Inf
##
## Scaled residuals:
##     Min      1Q  Median      3Q     Max
## -6.5881 -0.2417  0.0000  0.4735  5.6484
##
## Random effects:
##  Groups    Name        Variance Std.Dev.
##  exammonth (Intercept)   4.81    2.193
##  Residual              742.70   27.253
## Number of obs: 5053, groups:  exammonth, 12
##
## Fixed effects:
##               Estimate Std. Error t value
## (Intercept) 37.0877384  0.6419346   57.77
## age         -0.0038304  0.0002747  -13.94
## weight_KG    0.0017655  0.0003210    5.50
## height_CM   -0.0013128  0.0006298   -2.08
##
## Correlation of Fixed Effects:
##           (Intr) age    wgh_KG
## age       -0.062
## weight_KG  0.036 -0.065
## height_CM -0.160  0.270 -0.430
## convergence code: 0
## Gradient contains NAs
```

```
lmerNHANES_month_re <- data.frame(re=as.numeric(ranef(lmerNHANES_month)$exammonth[
,1]), se=as.numeric(se.ranef(lmerNHANES_month)$exammonth[,1]))
lmerNHANES_month_re$low <- lmerNHANES_month_re$re - 1.96 * lmerNHANES_month_re$se
lmerNHANES_month_re$high <- lmerNHANES_month_re$re + 1.96 * lmerNHANES_month_re$se
lmerNHANES_month_re$month <- factor(c("January","February","March","April","May","
June","July","August","September","October","November","December"),levels=c("Janua
ry","February","March","April","May","June","July","August","September","October",
"November","December"))
lmerNHANES_month_re$sex <- "men"
lmerNHANES_month_re$race <- "white"
lmerNHANES_month_re$period <- "NHANES"

ggplot(data=lmerNHANES_month_re,aes(x=month, y=re, color = "red")) + geom_point()+
geom_linerange(aes(ymin=low, ymax=high)) + labs(title="Month effect on temperature
in white men NHANES", x="", y="Temperature Difference, C", colour="", fill="") + t
heme(legend.position="none", axis.text.x = element_text(angle = 30, hjust = 1))
```

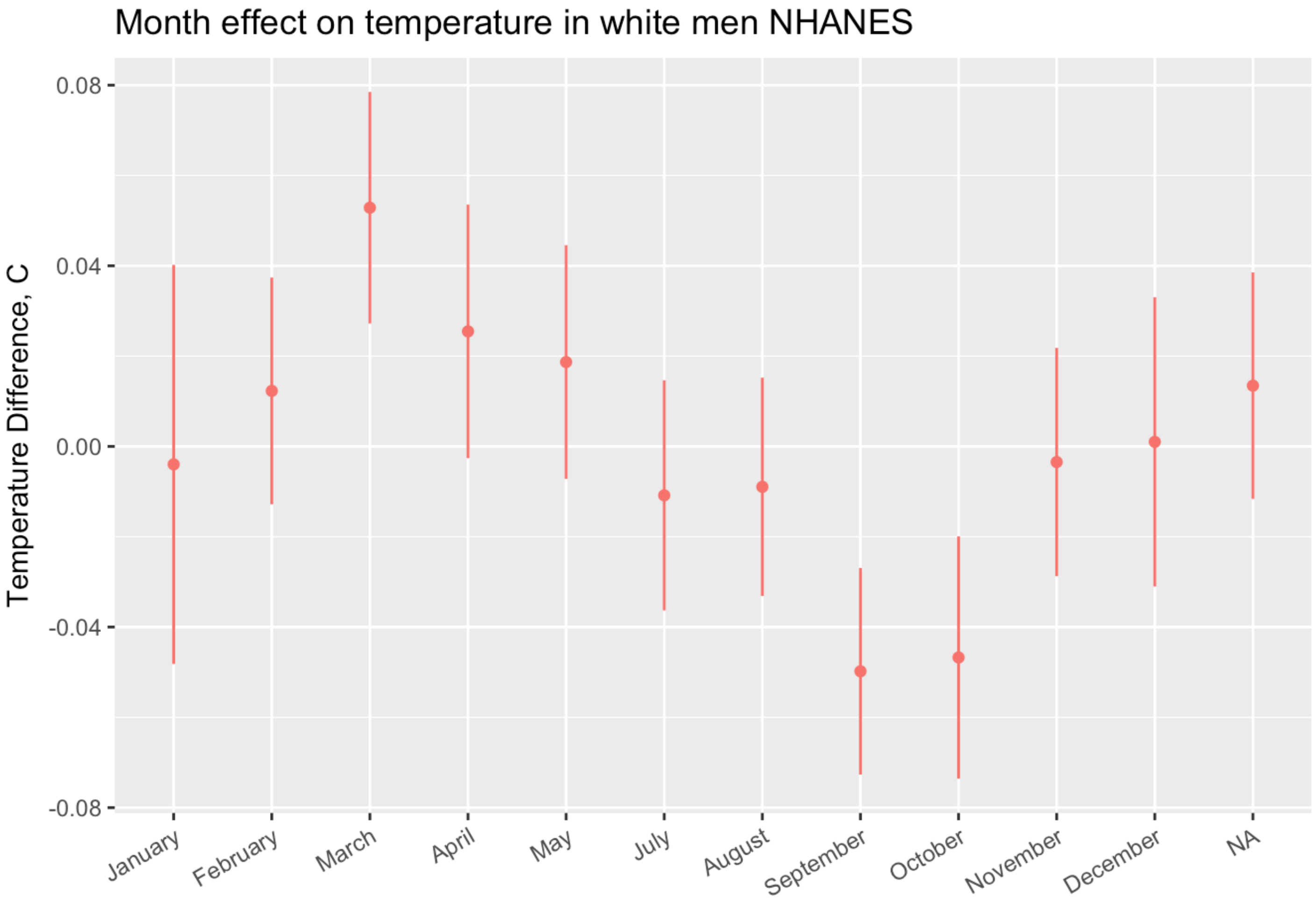


```
 lmNHANES_W_F_month <- lm(temp_C ~ age + weight_KG + height_CM +exammonth, data=da
taNHANES[dataNHANES$sex == "female" & dataNHANES$race == "white", ], weights=dataN
HANES$sample_weights[dataNHANES$sex=="female" & dataNHANES$race == "white"])
summary(lmNHANES_W_F_month)
```

```
##
## Call:
## lm(formula = temp_C ~ age + weight_KG + height_CM + exammonth,
##     data = dataNHANES[dataNHANES$sex == "female" & dataNHANES$race ==
##         "white", ], weights = dataNHANES$sample_weights[dataNHANES$sex ==
##         "female" & dataNHANES$race == "white"])
##
## Weighted Residuals:
##      Min       1Q   Median       3Q      Max
## -200.509   -6.401    0.000   10.007  159.919
##
## Coefficients:
##               Estimate Std. Error t value Pr(>|t|)
## (Intercept) 37.3072460  0.0952848 391.534  < 2e-16 ***
## age         -0.0044074  0.0002367 -18.616  < 2e-16 ***
## weight_KG    0.0007490  0.0002542   2.947  0.00322 **
## height_CM   -0.0017169  0.0005758  -2.982  0.00288 **
## exammonth2   0.0026780  0.0211307   0.127  0.89916
## exammonth3  -0.0041050  0.0215407  -0.191  0.84887
## exammonth4  -0.0279692  0.0223086  -1.254  0.20998
## exammonth5  -0.0057909  0.0209395  -0.277  0.78213
## exammonth6  -0.0067957  0.0211550  -0.321  0.74805
## exammonth7  -0.0306367  0.0214992  -1.425  0.15420
## exammonth8  -0.0540536  0.0210671  -2.566  0.01032 *
## exammonth9  -0.0432374  0.0210949  -2.050  0.04044 *
## exammonth10 -0.0594363  0.0211569  -2.809  0.00498 **
## exammonth11 -0.0512091  0.0210302  -2.435  0.01492 *
## exammonth12 -0.0693508  0.0232718  -2.980  0.00289 **
## ---
## Signif. codes:  0 '***' 0.001 '**' 0.01 '*' 0.05 '.' 0.1 ' ' 1
##
## Residual standard error: 25.07 on 6212 degrees of freedom
## Multiple R-squared:  0.06006,    Adjusted R-squared:  0.05794
## F-statistic: 28.35 on 14 and 6212 DF,  p-value: < 2.2e-16
```

```
lmerNHANES_month_F <-lmer(temp_C ~ age + weight_KG + height_CM + (1 | exammonth),
data=dataNHANES[dataNHANES$sex == "female" & dataNHANES$race == "white", ], weight
s=dataNHANES$sample_weights[dataNHANES$sex=="female" & dataNHANES$race == "white"]
)
summary(lmerNHANES_month_F)
```

```
## Linear mixed model fit by REML ['lmerMod']
## Formula: temp_C ~ age + weight_KG + height_CM + (1 | exammonth)
##    Data: 
## dataNHANES[dataNHANES$sex == "female" & dataNHANES$race == "white",      ]
## Weights: 
## dataNHANES$sample_weights[dataNHANES$sex == "female" & dataNHANES$race ==  
##     "white"]
## 
## REML criterion at convergence: Inf
## 
## Scaled residuals: 
##     Min      1Q  Median      3Q     Max 
## -8.8796 -0.2835  0.0000  0.4432  7.0820 
## 
## Random effects:
##  Groups    Name        Variance Std.Dev.
##  exammonth (Intercept)   1.703   1.305  
##  Residual              509.901  22.581  
## Number of obs: 7663, groups:  exammonth, 12
## 
## Fixed effects:
##               Estimate Std. Error t value
## (Intercept) 37.2780869  0.3859878   96.58
## age         -0.0044074  0.0002132  -20.67
## weight_KG    0.0007490  0.0002289    3.27
## height_CM   -0.0017169  0.0005186   -3.31
## 
## Correlation of Fixed Effects:
##           (Intr) age    wgh_KG
## age       -0.081              
## weight_KG  0.026 -0.189       
## height_CM -0.214  0.296 -0.277
## convergence code: 0
## Gradient contains NAs
```

```
lmerNHANES_month_F_re <- data.frame(re=as.numeric(ranef(lmerNHANES_month_F)$exammo
nth[,1]),se=as.numeric(se.ranef(lmerNHANES_month_F)$exammonth[,1]))
lmerNHANES_month_F_re$low <- lmerNHANES_month_F_re$re - 1.96 * lmerNHANES_month_F_
re$se
lmerNHANES_month_F_re$high <-lmerNHANES_month_F_re$re + 1.96 * lmerNHANES_month_F_
re$se
lmerNHANES_month_F_re$month <- factor(c("January","February","March","April","May"
,"June","July","August","September","October","November","December"),levels=c("Jan
uary","February","March","April","May","June","July","August","September","October
","November","December"))

lmerNHANES_month_F_re$sex <- "women"
lmerNHANES_month_F_re$race <- "white"
lmerNHANES_month_F_re$period <- "NHANES"
ggplot(data=lmerNHANES_month_F_re,aes(x=month, y=re, color = "red")) + geom_point(
) + geom_linerange(aes(ymin=low, ymax=high)) + labs(title="Month effect on tempera
ture in white women NHANES", x="", y="Temperature Difference, C", colour="", fill=
"") + theme(legend.position="none", axis.text.x = element_text(angle = 30, hjust =
1))
```

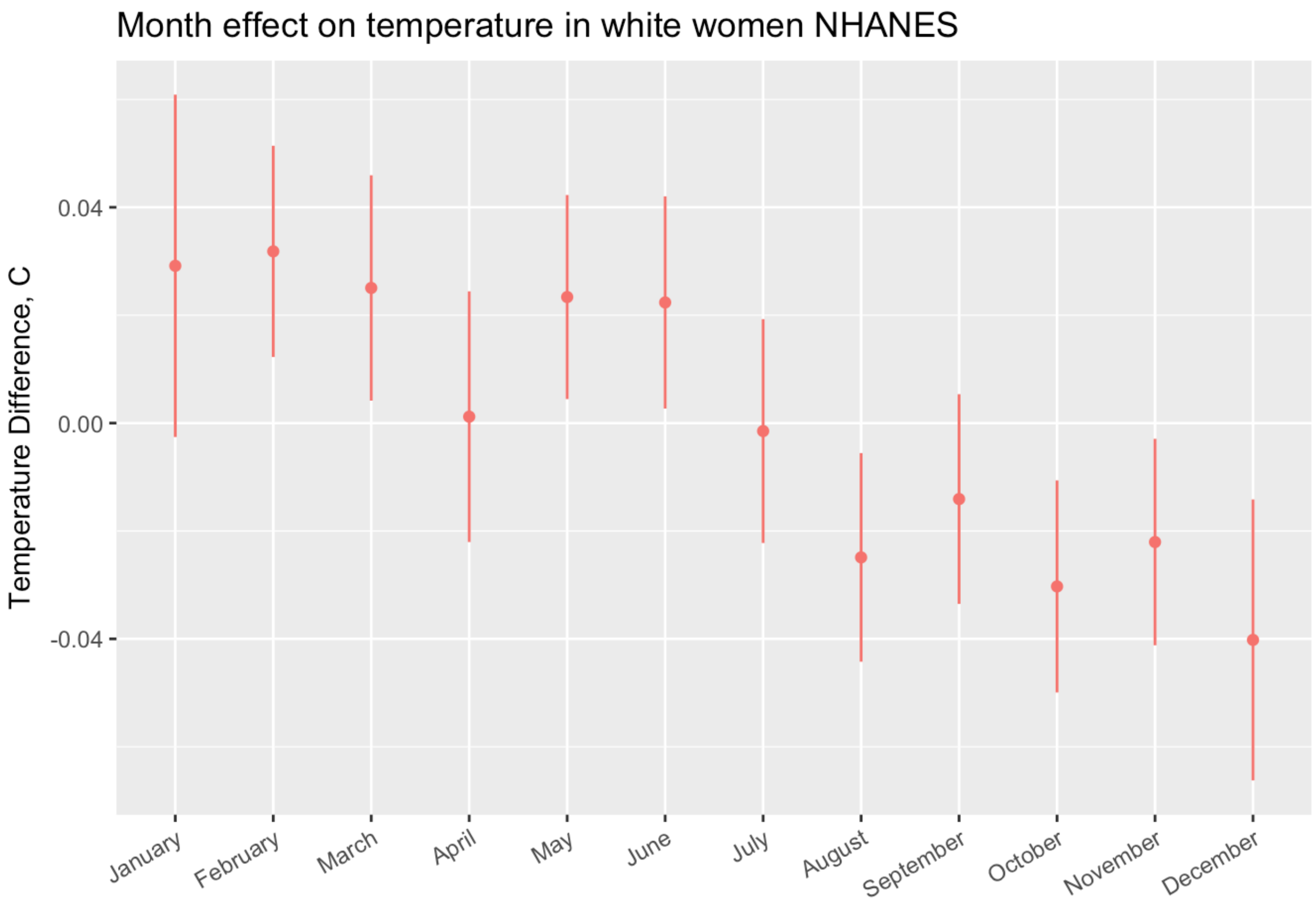

```
monthlyData <- rbind(lmerNHANES_month_F_re,lmerNHANES_month_re,lmerVeterans_month_
re,lmerST_data_month_re,lmerST_data_month_F_re)
monthlyData$sex <- factor(monthlyData$sex,levels=c("men","women"))
monthlyData$race <- factor(monthlyData$race,levels=c("white"))
monthlyData$period <- factor(monthlyData$period,levels=c("UAVCW","NHANES","STRIDE"
))

ggplot(data=monthlyData,aes(x=month, y=re)) + geom_point() + geom_linerange(aes(ym
in=low, ymax=high)) + labs(title="Month effect on temperature", x="", y="Temperatu
re Difference, C", colour="", fill="") + theme(legend.position="none", axis.text.x
= element_text(angle = 60, hjust = 1)) + facet_grid(sex ~ period)
```

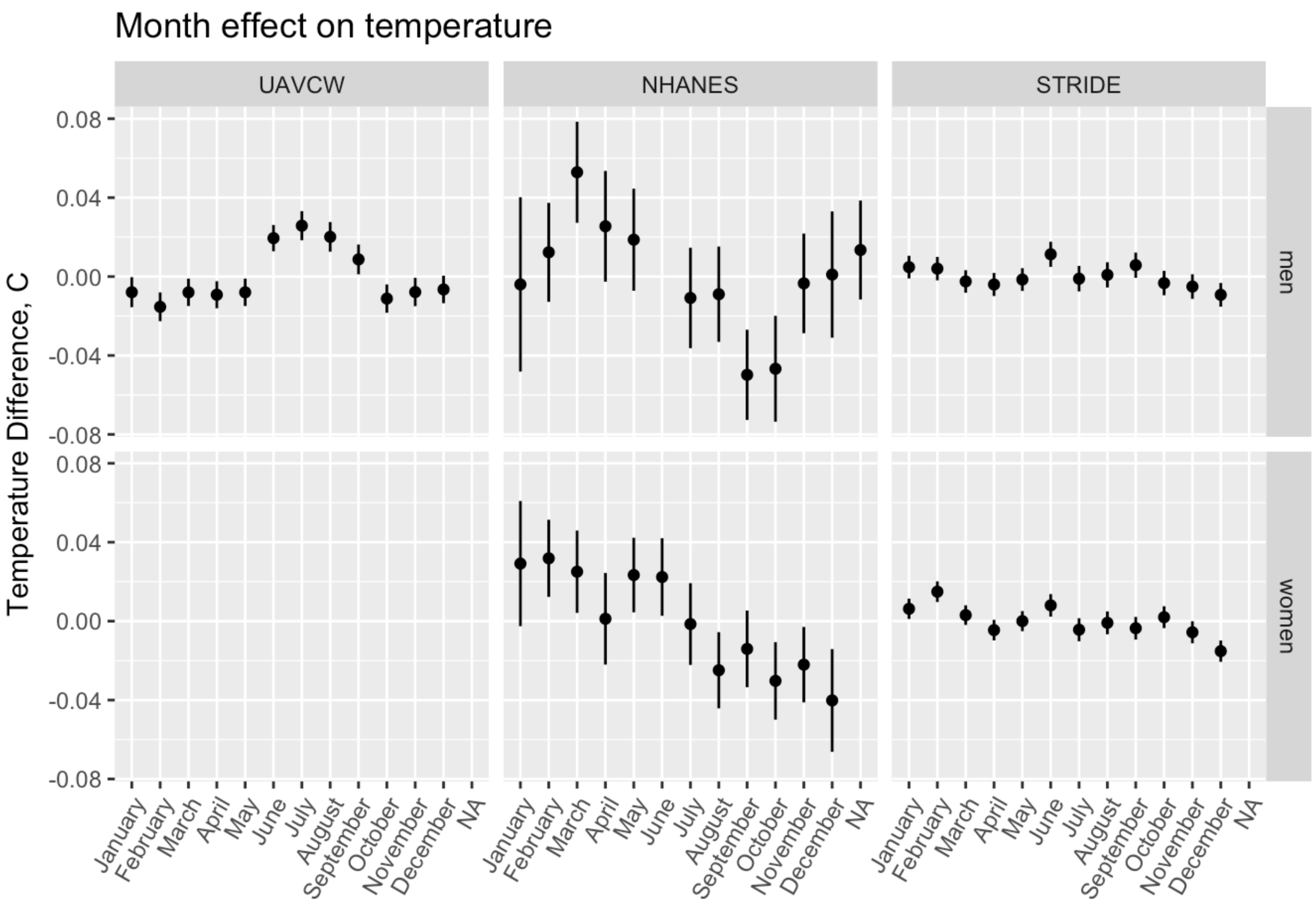

Analysis using both month and location (state)

```
lmVeterans_state <-lm (temp_C~ age + weightKG + heightCM + state_type  , data= dat
aVeterans [dataVeterans$race == "white",])
summary(lmVeterans_state)
```

```
##
## Call:
## lm(formula = temp_C ~ age + weightKG + heightCM + state_type,
##     data = dataVeterans[dataVeterans$race == "white", ])
##
## Residuals:
##      Min       1Q   Median       3Q      Max
## -1.84624 -0.09233 -0.01232  0.07629  2.07049
##
## Coefficients:
##                              Estimate Std. Error  t value Pr(>|t|)
## (Intercept)                 3.718e+01  2.994e-02 1242.035  < 2e-16 ***
## age                        -3.505e-03  1.231e-04  -28.468  < 2e-16 ***
## weightKG                   -2.178e-04  1.103e-04   -1.975   0.0483 *
## heightCM                   -2.055e-05  1.747e-04   -0.118   0.9064
## state_typecold             -2.023e-02  3.367e-03   -6.007 1.90e-09 ***
## state_typemoderately_cold -7.556e-03  3.118e-03   -2.423   0.0154 *
## state_typemoderate         -2.262e-02  5.667e-03   -3.992 6.56e-05 ***
## state_typewarm             -1.294e-02  1.004e-02   -1.289   0.1974
## state_typehot              -3.348e-02  2.962e-02   -1.130   0.2583
## ---
## Signif. codes:  0 '***' 0.001 '**' 0.01 '*' 0.05 '.' 0.1 ' ' 1
##
## Residual standard error: 0.2735 on 61927 degrees of freedom
##   (1097 observations deleted due to missingness)
## Multiple R-squared:  0.01383,    Adjusted R-squared:  0.0137
## F-statistic: 108.6 on 8 and 61927 DF,  p-value: < 2.2e-16
```

```
lmVeterans_month_state <-lm (temp_C~ age + weightKG + heightCM + exammonth + state
_type, data = dataVeterans [dataVeterans$race == "white",])
summary(lmVeterans_month_state)
```

```
##
## Call:
## lm(formula = temp_C ~ age + weightKG + heightCM + exammonth +
##     state_type, data = dataVeterans[dataVeterans$race == "white",
##     ])
##
## Residuals:
##      Min       1Q   Median       3Q      Max
## -1.83806 -0.09518 -0.01209  0.07870  2.04934
##
## Coefficients:
##                             Estimate Std. Error  t value Pr(>|t|)
## (Intercept)                3.717e+01  3.019e-02 1231.233  < 2e-16 ***
## age                       -3.500e-03  1.232e-04  -28.423  < 2e-16 ***
## weightKG                  -1.689e-04  1.104e-04   -1.530  0.12602
## heightCM                  -3.897e-05  1.746e-04   -0.223  0.82345
## exammonth2                -7.601e-03  5.616e-03   -1.354  0.17590
## exammonth3                -4.310e-04  5.480e-03   -0.079  0.93731
## exammonth4                -1.637e-03  5.451e-03   -0.300  0.76390
## exammonth5                 9.964e-05  5.480e-03    0.018  0.98549
## exammonth6                 2.853e-02  5.378e-03    5.304 1.13e-07 ***
## exammonth7                 3.562e-02  5.652e-03    6.303 2.94e-10 ***
## exammonth8                 2.977e-02  5.703e-03    5.219 1.80e-07 ***
## exammonth9                 1.605e-02  5.715e-03    2.808  0.00498 **
## exammonth10               -3.985e-03  5.568e-03   -0.716  0.47415
## exammonth11                3.849e-04  5.562e-03    0.069  0.94483
## exammonth12                1.210e-03  5.488e-03    0.220  0.82552
## state_typecold            -2.028e-02  3.366e-03   -6.024 1.71e-09 ***
## state_typemoderately_cold -7.563e-03  3.117e-03   -2.426  0.01525 *
## state_typemoderate        -2.277e-02  5.665e-03   -4.019 5.86e-05 ***
## state_typewarm            -1.295e-02  1.005e-02   -1.289  0.19750
## state_typehot             -3.532e-02  2.957e-02   -1.194  0.23233
## ---
## Signif. codes:  0 '***' 0.001 '**' 0.01 '*' 0.05 '.' 0.1 ' ' 1
##
## Residual standard error: 0.2731 on 61795 degrees of freedom
##   (1218 observations deleted due to missingness)
## Multiple R-squared:  0.01657,    Adjusted R-squared:  0.01627
## F-statistic: 54.81 on 19 and 61795 DF,  p-value: < 2.2e-16
```

Analysis of the ambient temperarure effect

```
lmVeterans_ambient <-lm (temp_C~ age + weightKG + heightCM + ambient_temp , data=
dataVeterans [dataVeterans$race == "white",])
summary(lmVeterans_ambient)
```

```
##
## Call:
## lm(formula = temp_C ~ age + weightKG + heightCM + ambient_temp,
##     data = dataVeterans[dataVeterans$race == "white", ])
##
## Residuals:
##      Min       1Q   Median       3Q      Max
## -1.68378 -0.07886  0.00993  0.07844  2.04801
##
## Coefficients:
##                Estimate Std. Error t value Pr(>|t|)
## (Intercept)   3.715e+01  4.047e-02 917.899   <2e-16 ***
## age          -3.806e-03  2.112e-04 -18.023   <2e-16 ***
## weightKG      1.830e-04  1.368e-04   1.338    0.181
## heightCM     -3.707e-05  2.296e-04  -0.161    0.872
## ambient_temp  1.307e-03  1.441e-04   9.071   <2e-16 ***
## ---
## Signif. codes:  0 '***' 0.001 '**' 0.01 '*' 0.05 '.' 0.1 ' ' 1
##
## Residual standard error: 0.2457 on 28119 degrees of freedom
##   (34909 observations deleted due to missingness)
## Multiple R-squared:  0.01461,    Adjusted R-squared:  0.01447
## F-statistic: 104.2 on 4 and 28119 DF,  p-value: < 2.2e-16
```

```
lmST_data_ambient <-lm(temp_C~ age_years + weight_KG + height_CM + ambient_temp, d
ata= ST_data [ST_data$GENDER == "Male" & ST_data$race == "White",])
summary(lmST_data_ambient)
```

```
##
## Call:
## lm(formula = temp_C ~ age_years + weight_KG + height_CM + ambient_temp,
##     data = ST_data[ST_data$GENDER == "Male" & ST_data$race ==
##         "White", ])
##
## Residuals:
##     Min      1Q  Median      3Q     Max
## -1.6353 -0.2030  0.0100  0.2118  2.3655
##
## Coefficients:
##                Estimate Std. Error  t value Pr(>|t|)
## (Intercept)   3.702e+01  2.618e-02 1413.781   <2e-16 ***
## age_years    -3.191e-03  6.886e-05  -46.346   <2e-16 ***
## weight_KG     1.254e-03  6.282e-05   19.966   <2e-16 ***
## height_CM    -1.954e-03  1.501e-04  -13.021   <2e-16 ***
## ambient_temp  4.072e-04  1.634e-04    2.492   0.0127 *
## ---
## Signif. codes:  0 '***' 0.001 '**' 0.01 '*' 0.05 '.' 0.1 ' ' 1
##
## Residual standard error: 0.3644 on 127108 degrees of freedom
##   (19414 observations deleted due to missingness)
## Multiple R-squared:  0.01834,    Adjusted R-squared:  0.01831
## F-statistic: 593.5 on 4 and 127108 DF,  p-value: < 2.2e-16
```

Then we present a plot of temperature broken down by birth decades the linear model that has birth decades as dummy variables (we only inlcude those birth cohorts that have more than 1000 measurements in each).

Figure 2: Temperature trends within birth cohorts of the UAVCW, 1860-1940 (black and white men).

```
# Minimum ages vary across the three cohorts due to the bias introduced by the sam
pling (it is impossible to be younger than 30, be born in 1820s, and be a veteran
of the Civil War). The values 52, 45, and 38 represent the cutoffs so that 99% of
observations in a respective cohort are older than this cutoff.

Veterans_birth_cohorts_C  <-ggplot(data=dataVeterans[ ((dataVeterans$age>52 & data
Veterans$age<80 & dataVeterans$birth_cohort== "1820s") | (dataVeterans$age>45 & da
taVeterans$age<80 & dataVeterans$birth_cohort== "1830s") | (dataVeterans$age>38 &
dataVeterans$age<80 & dataVeterans$birth_cohort== "1840s"))  & dataVeterans$race =
= "white", ], aes(age, temp_C, group=birth_cohort, colour= birth_cohort))   + geom
_smooth()

Veterans_birth_cohorts_C + theme(legend.position="bottom", axis.text=element_text(
size=14), axis.title=element_text(size=14), legend.text=element_text(size=14)) + l
abs(x= "Age, years", y = "Temperature,°C", colour = "Birth decade" ) + scale_colour
_manual(values = plot_colors[c(2,3,1 )]) #+ylim(36.7,37)
```

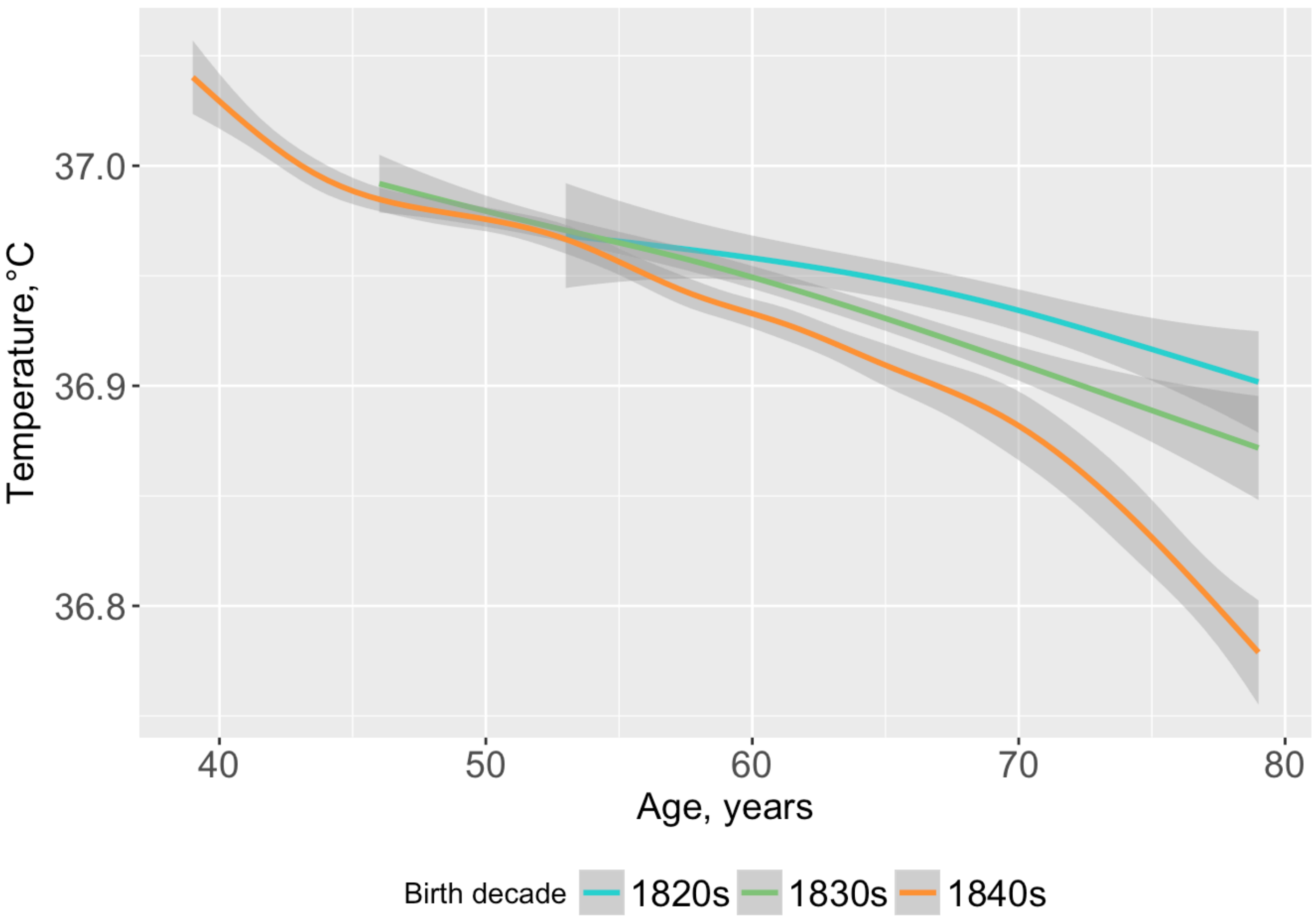


```
lmBirthcohorts <-lm(temp_C ~ age + weightKG + heightCM + race + birth_cohort, data
 =dataVeterans [dataVeterans$birth_cohort %in%c ( "1820s", "1830s",  "1840s"), ])
 summary (lmBirthcohorts)
```

```
##
## Call:
## lm(formula = temp_C ~ age + weightKG + heightCM + race + birth_cohort,
##     data = dataVeterans[dataVeterans$birth_cohort %in% c("1820s",
##         "1830s", "1840s"), ])
##
## Residuals:
##      Min       1Q   Median       3Q      Max
## -1.83583 -0.08272 -0.00879  0.07162  2.05343
##
## Coefficients:
##                      Estimate Std. Error  t value Pr(>|t|)
## (Intercept)         3.718e+01  2.525e-02 1472.917  < 2e-16 ***
## age                -3.550e-03  1.186e-04  -29.925  < 2e-16 ***
## weightKG           -2.001e-04  9.346e-05   -2.141   0.0323 *
## heightCM            4.524e-05  1.452e-04    0.312   0.7553
## raceblack          -1.874e-02  2.133e-03   -8.785  < 2e-16 ***
## birth_cohort1830s -1.766e-02  3.358e-03   -5.257 1.47e-07 ***
## birth_cohort1840s -3.109e-02  3.467e-03   -8.966  < 2e-16 ***
## ---
## Signif. codes:  0 '***' 0.001 '**' 0.01 '*' 0.05 '.' 0.1 ' ' 1
##
## Residual standard error: 0.2607 on 82569 degrees of freedom
## Multiple R-squared:  0.01269,    Adjusted R-squared:  0.01261
## F-statistic: 176.8 on 6 and 82569 DF,  p-value: < 2.2e-16
```

And we present predicted temperature for men at age 30, weight 70 kg, and hieght 170 cm for each of these decades in a table

```
 predict_dataframe <- data.frame(age=30, weightKG=70, heightCM = 170, race= "white
", birth_cohort="1820s")
 round(predict (lmBirthcohorts , predict_dataframe , interval="confidence"), 2)
```

```
##     fit   lwr   upr
## 1 37.07 37.06 37.08
```

```
predict_dataframe <- data.frame(age=30, weightKG=70, heightCM = 170,race= "white",
birth_cohort="1830s")
 round(predict (lmBirthcohorts , predict_dataframe , interval="confidence"),2)
```

```
##     fit   lwr   upr
## 1 37.05 37.05 37.06
```

As as a final piece of the analysis we present both table with coeficient and plots of the polynomial function of a birth year. We have two separate models for men and women, and we include all races. But on the graph we plot predicted values for white men and white women.

```
combined_subset <- combinedData$sex=="male" & combinedData$race =="white" & !is.na
(combinedData$race) & !is.na(combinedData$height_CM) & !is.na(combinedData$weight_
KG) & !is.na(combinedData$sex) & combinedData$sample_weights > 0

combined_subset_f <- combinedData$sex=="female" & combinedData$race =="white" & !i
s.na(combinedData$race) & !is.na(combinedData$height_CM) & !is.na(combinedData$wei
ght_KG) & !is.na(combinedData$sex) & combinedData$sample_weights > 0

linear <-lm(temp_C ~ age + weight_KG + height_CM + I(birth_year-1800),  data=combi
nedData[combined_subset, ] )
summary(linear)
```

```
##
## Call:
## lm(formula = temp_C ~ age + weight_KG + height_CM + I(birth_year -
##     1800), data = combinedData[combined_subset, ])
##
## Residuals:
##      Min       1Q   Median       3Q      Max
## -1.86352 -0.17577 -0.00258  0.18360  2.36636
##
## Coefficients:
##                        Estimate Std. Error t value Pr(>|t|)
## (Intercept)           3.760e+01  1.944e-02 1934.46   <2e-16 ***
## age                  -5.989e-03  5.983e-05 -100.10   <2e-16 ***
## weight_KG             9.663e-04  5.256e-05   18.38   <2e-16 ***
## height_CM            -1.492e-03  1.148e-04  -13.00   <2e-16 ***
## I(birth_year - 1800) -2.981e-03  1.660e-05 -179.63   <2e-16 ***
## ---
## Signif. codes:  0 '***' 0.001 '**' 0.01 '*' 0.05 '.' 0.1 ' ' 1
##
## Residual standard error: 0.3365 on 194075 degrees of freedom
## Multiple R-squared:  0.1938, Adjusted R-squared:  0.1938
## F-statistic: 1.166e+04 on 4 and 194075 DF,  p-value: < 2.2e-16
```

```
linear_f <-lm(temp_C ~ age + weight_KG + height_CM + I(birth_year-1800),  data=com
binedData[combined_subset_f, ] )
summary(linear_f)
```

```
##
## Call:
## lm(formula = temp_C ~ age + weight_KG + height_CM + I(birth_year -
##     1800), data = combinedData[combined_subset_f, ])
##
## Residuals:
##      Min       1Q   Median       3Q      Max
## -1.69003 -0.20374  0.00141  0.20557  2.34815
##
## Coefficients:
##                        Estimate Std. Error  t value Pr(>|t|)
## (Intercept)           3.765e+01  2.898e-02 1299.039   <2e-16 ***
## age                  -6.613e-03  1.130e-04  -58.513   <2e-16 ***
## weight_KG             7.886e-04  4.955e-05   15.916   <2e-16 ***
## height_CM            -1.202e-03  1.236e-04   -9.724   <2e-16 ***
## I(birth_year - 1800) -2.866e-03  1.060e-04  -27.028   <2e-16 ***
## ---
## Signif. codes:  0 '***' 0.001 '**' 0.01 '*' 0.05 '.' 0.1 ' ' 1
##
## Residual standard error: 0.3566 on 184690 degrees of freedom
## Multiple R-squared:  0.03232,    Adjusted R-squared:  0.0323
## F-statistic:  1542 on 4 and 184690 DF,  p-value: < 2.2e-16
```

```
pred_df <- data.frame(age=rep(30,201), birth_year=seq(1800,2000), weight_KG = rep(
70,201), height_CM = rep(170,201), race=rep("white",201), sex=rep("male",201))
pred_df$mean <- (predict(linear, pred_df,interval="confidence"))[,1]
pred_df$ci025 <- (predict(linear, pred_df,interval="confidence"))[,2]
pred_df$ci975 <- (predict(linear, pred_df,interval="confidence"))[,3]

pred_df_f <- data.frame(age=rep(30,111), birth_year=seq(1890,2000), weight_KG = re
p(70,111), height_CM = rep(170,111), race=rep("white",111), sex=rep("female",111))
pred_df_f$mean <- (predict(linear_f, pred_df_f,interval="confidence"))[,1]
pred_df_f$ci025 <- (predict(linear_f, pred_df_f,interval="confidence"))[,2]
pred_df_f$ci975 <- (predict(linear_f, pred_df_f,interval="confidence"))[,3]


predictions <- rbind(pred_df, pred_df_f)


ggplot() + geom_ribbon(data = pred_df, aes(birth_year, ymin=ci025,ymax=ci975),fill
="blue",alpha=0.3) + geom_line(data = pred_df, aes(birth_year, mean),colour="blue"
) + geom_ribbon(data = pred_df_f, aes(birth_year, ymin=ci025,ymax=ci975),alpha=0.3
,fill="red") + geom_line(data = pred_df_f, aes(birth_year, mean),colour="red") + l
abs(x= "Year of birth", y = "Temperature,°C") + scale_fill_identity(name = 'the fil
l', guide = 'legend',labels = c('m1')) + scale_colour_manual(name = 'the colour',
values =c("blue"="blue","red"="red"), labels = c("Men","Women")) +ylim(36,37.5)
```

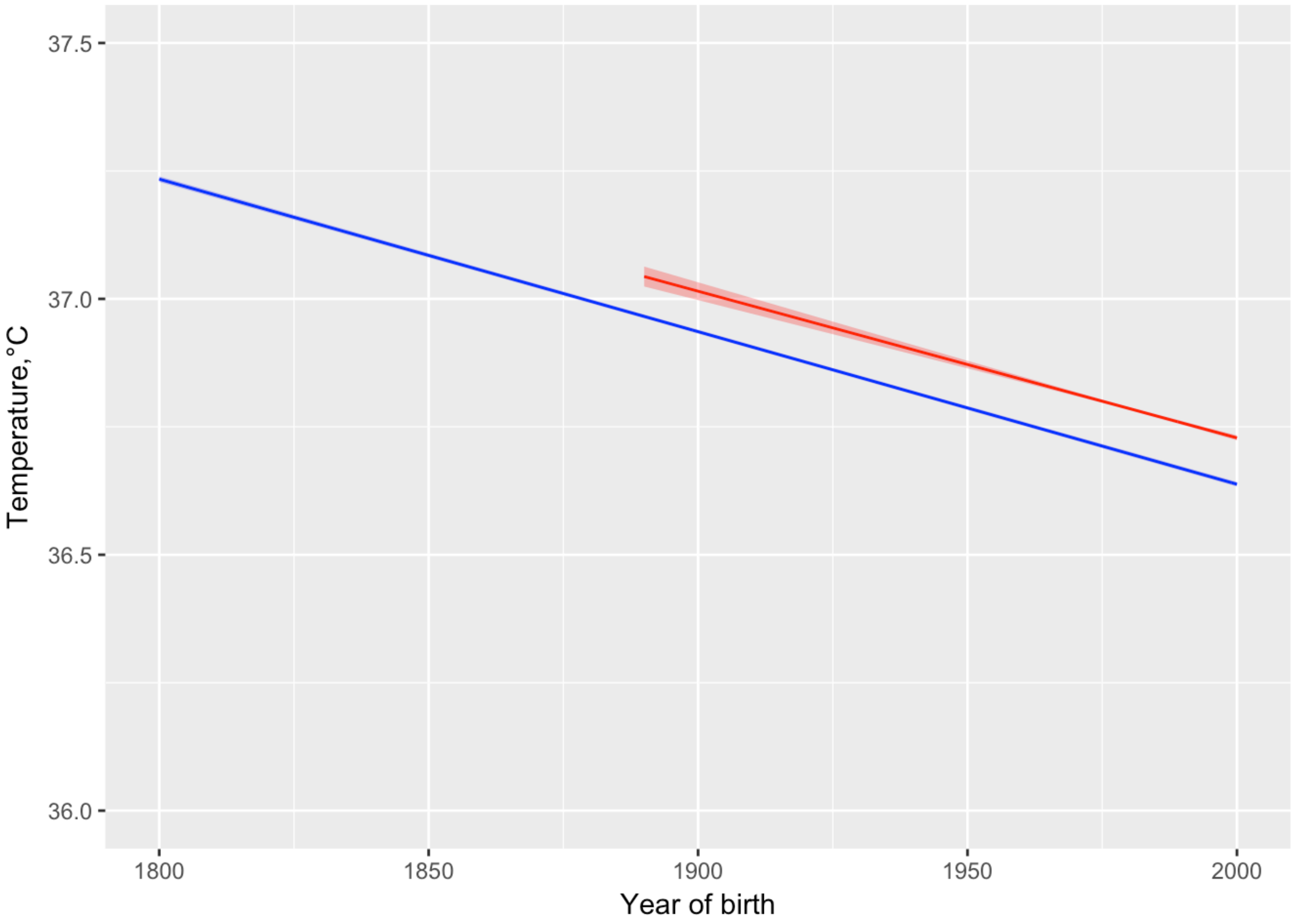


```
predictions$mean[predictions$birth_year==1800 & predictions$sex == "male"]
```

```
## [1] 37.23402
```

```
 predictions$mean[predictions$birth_year==2000 & predictions$sex == "male"]
```

```
## [1] 36.63782
```

```
(predictions$mean[predictions$birth_year==1800 & predictions$sex == "male"]- predi
ctions$mean[predictions$birth_year==2000 & predictions$sex == "male"])
```

```
## [1] 0.5961985
```

```
 predictions$mean[predictions$birth_year==1890 & predictions$sex == "female"]
```

```
## [1] 37.04365
```

```
predictions$mean[predictions$birth_year==2000 & predictions$sex == "female"]
```

```
## [1] 36.72841
```

```
(predictions$mean[predictions$birth_year==1890 & predictions$sex == "female"]- pre
dictions$mean[predictions$birth_year==2000 & predictions$sex == "female"])
```

```
## [1] 0.3152466
```

```
combined_subset_b <- combinedData$sex=="male" & combinedData$race =="black" & !is.
na(combinedData$race) & !is.na(combinedData$height_CM) & !is.na(combinedData$weigh
t_KG) & !is.na(combinedData$sex) & combinedData$sample_weights > 0

linear_b <-lm(temp_C ~ age + weight_KG + height_CM + I(birth_year-1800),  data=com
binedData[combined_subset_b, ] )
summary(linear_b)
```

```
##
## Call:
## lm(formula = temp_C ~ age + weight_KG + height_CM + I(birth_year -
##     1800), data = combinedData[combined_subset_b, ])
##
## Residuals:
##      Min       1Q   Median       3Q      Max
## -1.52876 -0.13884  0.00989  0.12627  2.36480
##
## Coefficients:
##                        Estimate Std. Error t value Pr(>|t|)
## (Intercept)           3.746e+01  3.925e-02 954.413  < 2e-16 ***
## age                  -5.357e-03  1.382e-04 -38.773  < 2e-16 ***
## weight_KG             4.199e-04  1.103e-04   3.807 0.000141 ***
## height_CM            -7.077e-04  2.407e-04  -2.940 0.003279 **
## I(birth_year - 1800) -3.225e-03  3.515e-05 -91.738  < 2e-16 ***
## ---
## Signif. codes:  0 '***' 0.001 '**' 0.01 '*' 0.05 '.' 0.1 ' ' 1
##
## Residual standard error: 0.3033 on 37026 degrees of freedom
## Multiple R-squared:  0.2714, Adjusted R-squared:  0.2713
## F-statistic:  3448 on 4 and 37026 DF,  p-value: < 2.2e-16
```

```
combined_subset_b_f <- combinedData$sex=="female" & combinedData$race =="black" &
!is.na(combinedData$race) & !is.na(combinedData$height_CM) & !is.na(combinedData$w
eight_KG) & !is.na(combinedData$sex) & combinedData$sample_weights > 0

linear_b_f <-lm(temp_C ~ age + weight_KG + height_CM + I(birth_year-1800),  data=c
ombinedData[combined_subset_b_f, ] )
summary(linear_b_f)
```

```
##
## Call:
## lm(formula = temp_C ~ age + weight_KG + height_CM + I(birth_year -
##     1800), data = combinedData[combined_subset_b_f, ])
##
## Residuals:
##      Min       1Q   Median       3Q      Max
## -1.66626 -0.20902  0.02414  0.23384  2.40442
##
## Coefficients:
##                        Estimate Std. Error t value Pr(>|t|)
## (Intercept)          38.0355388  0.0677383 561.507  < 2e-16 ***
## age                  -0.0086761  0.0002544 -34.103  < 2e-16 ***
## weight_KG             0.0004332  0.0001109   3.908 9.34e-05 ***
## height_CM            -0.0019213  0.0003084  -6.229 4.74e-10 ***
## I(birth_year - 1800) -0.0039824  0.0002343 -16.999  < 2e-16 ***
## ---
## Signif. codes:  0 '***' 0.001 '**' 0.01 '*' 0.05 '.' 0.1 ' ' 1
##
## Residual standard error: 0.3709 on 31687 degrees of freedom
## Multiple R-squared:  0.05105,    Adjusted R-squared:  0.05093
## F-statistic: 426.1 on 4 and 31687 DF,  p-value: < 2.2e-16
```

```
pred_df_b <- data.frame(age=rep(30,201), birth_year=seq(1800,2000), weight_KG = re
p(70,201), height_CM = rep(170,201), race=rep("black",201), sex=rep("male",201))
pred_df_b$mean <- (predict(linear_b, pred_df_b,interval="confidence"))[,1]
pred_df_b$ci025 <- (predict(linear_b, pred_df_b,interval="confidence"))[,2]
pred_df_b$ci975 <- (predict(linear_b, pred_df_b,interval="confidence"))[,3]

pred_df_b_f <- data.frame(age=rep(30,111), birth_year=seq(1890,2000), weight_KG =
rep(70,111), height_CM = rep(170,111), race=rep("black",111), sex=rep("female",111
))
pred_df_b_f$mean <- (predict(linear_b_f, pred_df_b_f,interval="confidence"))[,1]
pred_df_b_f$ci025 <- (predict(linear_b_f, pred_df_b_f,interval="confidence"))[,2]
pred_df_b_f$ci975 <- (predict(linear_b_f, pred_df_b_f,interval="confidence"))[,3]


predictions_b <- rbind(pred_df_b, pred_df_b_f)

predictions_full <- rbind(predictions,predictions_b)

ggplot(data = predictions_full) + geom_ribbon(aes(birth_year, ymin=ci025,ymax=ci97
5,fill=sex),alpha=0.3) + geom_line(aes(birth_year, mean,colour=sex),show.legend=FA
LSE) + scale_colour_manual(values = plot_colors[c(9,8)]) + scale_fill_manual(value
s = plot_colors [c(9,8 )], name="Sex",labels=c("Men","Women")) + facet_grid(. ~ ra
ce ) + labs(x= "Year of birth", y = "Temperature,°C") +ylim(36,37.5) + theme(legend
.position="bottom",axis.title=element_text(size=14,face="bold"), legend.title=elem
ent_text(size=14), legend.text=element_text(size=14))
```

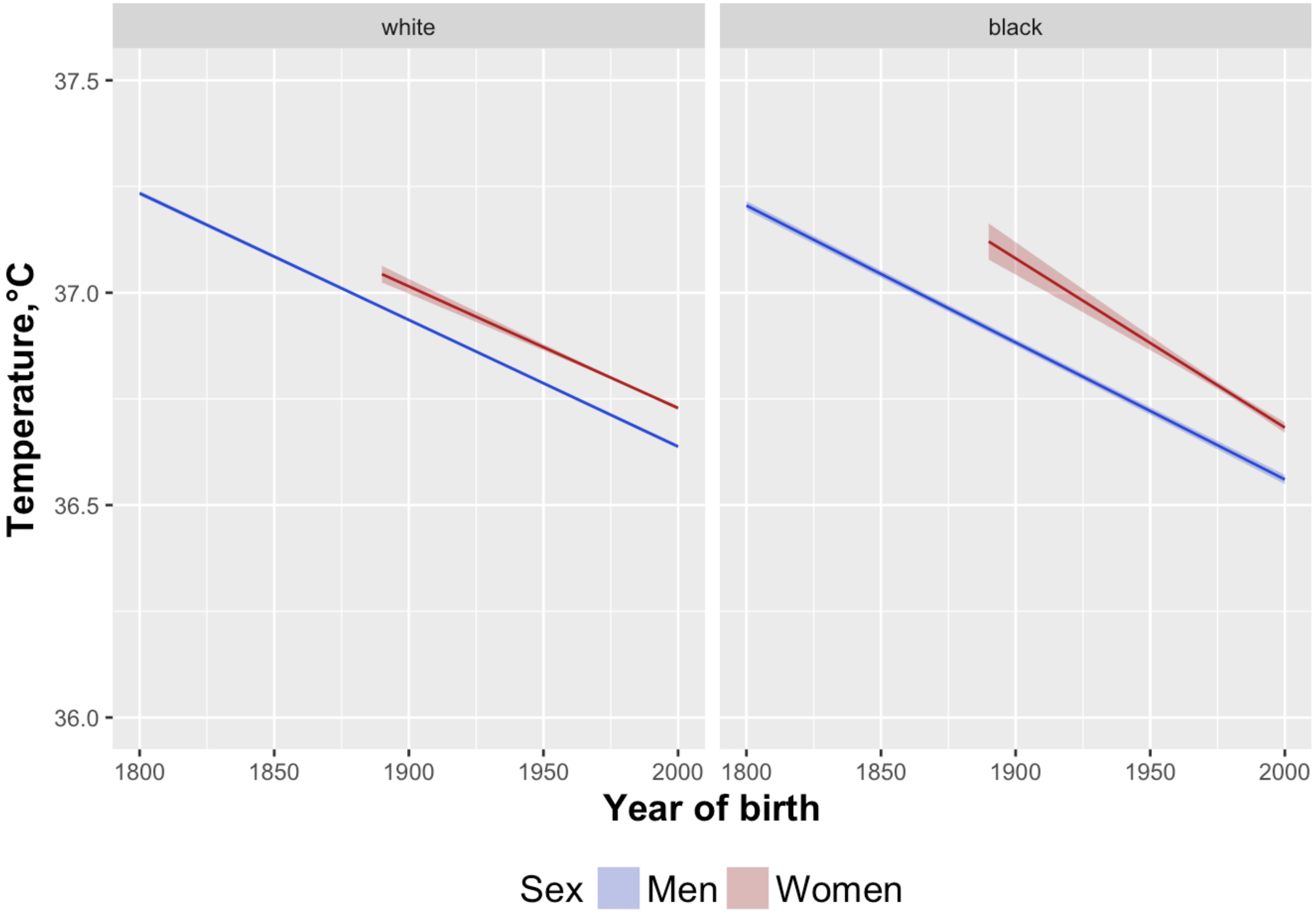

Figure 3: Model of mean body temperature over time in three cohorts by birth year (black and white race groups).

```
predictions_b$mean[predictions_b$birth_year==1800 & predictions_b$sex == "male"]
```

```
## [1] 37.20526
```

```
 predictions_b$mean[predictions_b$birth_year==2000 & predictions_b$sex == "male"]
```

```
## [1] 36.56034
```

```
(predictions_b$mean[predictions_b$birth_year==1800 & predictions_b$sex == "male"]-
predictions_b$mean[predictions_b$birth_year==2000 & predictions_b$sex == "male"])
```

```
## [1] 0.6449192
```

```
 predictions_b$mean[predictions_b$birth_year==1890 & predictions_b$sex == "female"
]
```

```
## [1] 37.12055
```

```
 predictions_b$mean[predictions_b$birth_year==2000 & predictions_b$sex == "female"
]
```

```
## [1] 36.68249
```

```
(predictions_b$mean[predictions_b$birth_year==1890 & predictions_b$sex == "female"
]- predictions_b$mean[predictions_b$birth_year==2000 & predictions_b$sex == "femal
e"])
```

```
## [1] 0.4380604
```

We do same analysis controling for the time of day of the temperatre measurement to chech for robustness of the result. For the UAVCW data we imputed the value of 12:00 PM (noon)

```
linear_HR <-lm(temp_C ~ age + weight_KG + height_CM + time_HR_imputed+ I(birth_yea
r-1800),  data=combinedData[combined_subset, ] )
summary(linear_HR)
```

```
##
## Call:
## lm(formula = temp_C ~ age + weight_KG + height_CM + time_HR_imputed +
##     I(birth_year - 1800), data = combinedData[combined_subset,
##     ])
##
## Residuals:
##      Min       1Q   Median       3Q      Max
## -1.86038 -0.17344 -0.00295  0.18242  2.40728
##
## Coefficients:
##                        Estimate Std. Error t value Pr(>|t|)
## (Intercept)           3.732e+01  1.973e-02 1891.03   <2e-16 ***
## age                  -5.793e-03  5.929e-05  -97.71   <2e-16 ***
## weight_KG             9.764e-04  5.201e-05   18.77   <2e-16 ***
## height_CM            -1.419e-03  1.136e-04  -12.49   <2e-16 ***
## time_HR_imputed       2.141e-02  3.340e-04   64.08   <2e-16 ***
## I(birth_year - 1800) -2.960e-03  1.643e-05 -180.23   <2e-16 ***
## ---
## Signif. codes:  0 '***' 0.001 '**' 0.01 '*' 0.05 '.' 0.1 ' ' 1
##
## Residual standard error: 0.333 on 194074 degrees of freedom
## Multiple R-squared:  0.2105, Adjusted R-squared:  0.2105
## F-statistic: 1.035e+04 on 5 and 194074 DF,  p-value: < 2.2e-16
```

```
linear_f_HR <-lm(temp_C ~ age + weight_KG + height_CM +time_HR_imputed + I(birth_y
ear-1800),  data=combinedData[combined_subset_f, ] )
summary(linear_f_HR)
```

```
##
## Call:
## lm(formula = temp_C ~ age + weight_KG + height_CM + time_HR_imputed +
##     I(birth_year - 1800), data = combinedData[combined_subset_f,
##     ])
##
## Residuals:
##      Min       1Q   Median       3Q      Max
## -1.70048 -0.20146  0.00112  0.20383  2.38394
##
## Coefficients:
##                        Estimate Std. Error  t value Pr(>|t|)
## (Intercept)           3.733e+01  2.909e-02 1283.292   <2e-16 ***
## age                  -6.220e-03  1.120e-04  -55.550   <2e-16 ***
## weight_KG             8.021e-04  4.901e-05   16.365   <2e-16 ***
## height_CM            -1.175e-03  1.223e-04   -9.607   <2e-16 ***
## time_HR_imputed       1.983e-02  3.109e-04   63.782   <2e-16 ***
## I(birth_year - 1800) -2.543e-03  1.050e-04  -24.214   <2e-16 ***
## ---
## Signif. codes:  0 '***' 0.001 '**' 0.01 '*' 0.05 '.' 0.1 ' ' 1
##
## Residual standard error: 0.3527 on 184689 degrees of freedom
## Multiple R-squared:  0.05318,    Adjusted R-squared:  0.05315
## F-statistic:  2075 on 5 and 184689 DF,  p-value: < 2.2e-16
```

```
pred_df_HR <- data.frame(age=rep(30,201), birth_year=seq(1800,2000), weight_KG = r
ep(70,201), height_CM = rep(170,201), race=rep("white",201), sex=rep("male",201),
time_HR_imputed=12)
pred_df_HR$mean <- (predict(linear_HR, pred_df_HR,interval="confidence"))[,1]
pred_df_HR$ci025 <- (predict(linear_HR, pred_df_HR,interval="confidence"))[,2]
pred_df_HR$ci975 <- (predict(linear_HR, pred_df_HR,interval="confidence"))[,3]

pred_df_f_HR <- data.frame(age=rep(30,111), birth_year=seq(1890,2000), weight_KG =
rep(70,111), height_CM = rep(170,111), race=rep("white",111), sex=rep("female",111
),time_HR_imputed=12)
pred_df_f_HR$mean <- (predict(linear_f_HR, pred_df_f_HR,interval="confidence"))[,1
]
pred_df_f_HR$ci025 <- (predict(linear_f_HR, pred_df_f_HR,interval="confidence"))[,
2]
pred_df_f_HR$ci975 <- (predict(linear_f_HR, pred_df_f_HR,interval="confidence"))[,
3]


predictions_HR <- rbind(pred_df_HR, pred_df_f_HR)


ggplot() + geom_ribbon(data = pred_df_HR, aes(birth_year, ymin=ci025,ymax=ci975),f
ill="blue",alpha=0.3) + geom_line(data = pred_df_HR, aes(birth_year, mean),colour=
"blue") + geom_ribbon(data = pred_df_f_HR, aes(birth_year, ymin=ci025,ymax=ci975),
alpha=0.3,fill="red") + geom_line(data = pred_df_f_HR, aes(birth_year, mean),colou
r="red") + labs(x= "Year of birth", y = "Temperature,°C") + scale_fill_identity(nam
e = 'the fill', guide = 'legend',labels = c('m1')) + scale_colour_manual(name = 't
he colour', values =c("blue"="blue","red"="red"), labels = c("Men","Women")) +ylim
(36,37.5)
```

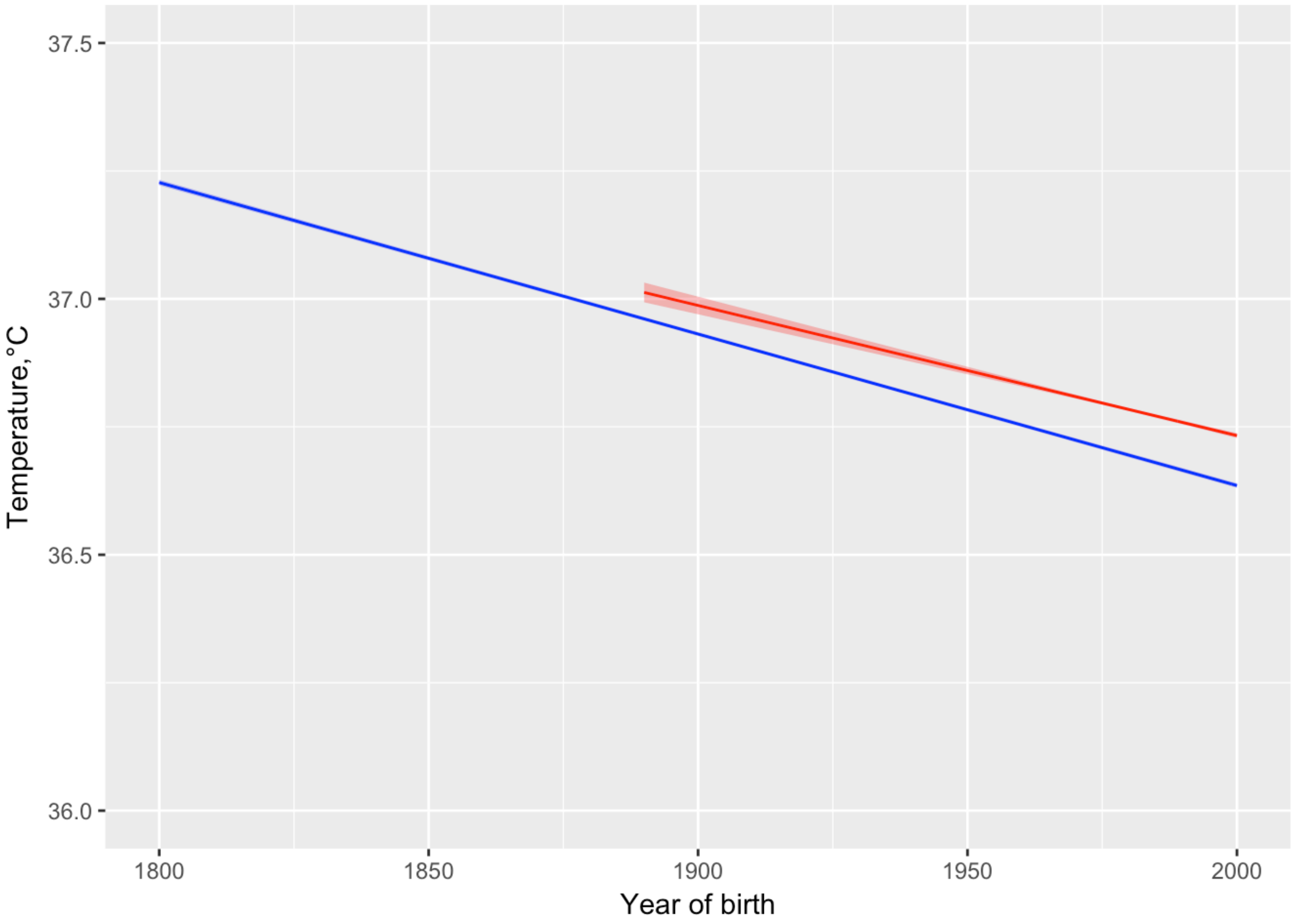

Figure 2S: Model of mean body temperature over time in three cohorts by birth year controlling for the time of the day of the temperature measurement (white men and women)

```
predictions_HR$mean[predictions_HR$birth_year==1800 & predictions_HR$sex == "male"
]
```

```
## [1] 37.22734
```

```
 predictions_HR$mean[predictions_HR$birth_year==2000 & predictions_HR$sex == "male
"]
```

```
## [1] 36.63526
```

```
(predictions_HR$mean[predictions_HR$birth_year==1800 & predictions_HR$sex == "male
"]- predictions_HR$mean[predictions_HR$birth_year==2000 & predictions_HR$sex == "m
ale"])
```

```
## [1] 0.5920851
```

```
 predictions_HR$mean[predictions_HR$birth_year==1890 & predictions_HR$sex == "fema
le"]
```

```
## [1] 37.01257
```

```
 predictions_HR$mean[predictions_HR$birth_year==2000 & predictions_HR$sex == "fema
le"]
```

```
## [1] 36.73288
```

```
(predictions_HR$mean[predictions_HR$birth_year==1890 & predictions_HR$sex == "fema
le"]- predictions_HR$mean[predictions_HR$birth_year==2000 & predictions_HR$sex ==
"female"])
```

```
## [1] 0.2796887
```

Here we make temperature predictions for UAVCW that have (had) various infections.

```
lmVets_inf <- lm(temp_C ~ age + weightKG+ heightCM + cholera + cystitis + dengue +
fever_unspec +gastro + goiter +  hepatitis + influenza + malaria + nephritis + pne
umonia + scarlet_fever + sepsis + smallpox  + syphilis+ tb, data=dataVeterans)

summary(lmVets_inf)
```

```
##
## Call:
## lm(formula = temp_C ~ age + weightKG + heightCM + cholera + cystitis +
##     dengue + fever_unspec + gastro + goiter + hepatitis + influenza +
##     malaria + nephritis + pneumonia + scarlet_fever + sepsis +
##     smallpox + syphilis + tb, data = dataVeterans)
##
## Residuals:
##      Min       1Q   Median       3Q      Max
## -1.82804 -0.08226 -0.00646  0.06839  2.06378
##
## Coefficients:
##                     Estimate Std. Error  t value Pr(>|t|)
## (Intercept)        3.711e+01  2.433e-02 1525.593  < 2e-16 ***
## age               -2.995e-03  1.027e-04  -29.159  < 2e-16 ***
## weightKG          -1.719e-04  9.253e-05   -1.858   0.0632 .
## heightCM           1.010e-04  1.428e-04    0.707   0.4796
## choleraTRUE       -3.534e-02  9.231e-02   -0.383   0.7019
## cystitisTRUE       7.610e-03  7.315e-03    1.040   0.2982
## dengueTRUE        -7.143e-02  4.415e-02   -1.618   0.1057
## fever_unspecTRUE  -2.626e-01  5.412e-02   -4.853 1.22e-06 ***
## gastroTRUE         1.307e-02  8.579e-03    1.524   0.1276
## goiterTRUE        -3.132e-03  3.145e-02   -0.100   0.9207
## hepatitisTRUE      4.710e-02  5.332e-02    0.883   0.3771
## influenzaTRUE      3.056e-02  3.145e-02    0.972   0.3312
## malariaTRUE        6.425e-03  5.865e-03    1.095   0.2733
## nephritisTRUE      7.149e-03  1.847e-02    0.387   0.6987
## pneumoniaTRUE      3.103e-02  1.573e-02    1.972   0.0486 *
## scarlet_feverTRUE -1.008e-02  8.257e-02   -0.122   0.9028
## sepsisTRUE         1.054e-02  3.588e-02    0.294   0.7690
## smallpoxTRUE       6.945e-03  1.334e-02    0.521   0.6027
## syphilisTRUE       2.142e-02  1.214e-02    1.764   0.0777 .
## tbTRUE             1.982e-01  9.836e-03   20.152  < 2e-16 ***
## ---
## Signif. codes:  0 '***' 0.001 '**' 0.01 '*' 0.05 '.' 0.1 ' ' 1
##
## Residual standard error: 0.2611 on 83880 degrees of freedom
## Multiple R-squared:  0.01549,    Adjusted R-squared:  0.01526
## F-statistic: 69.45 on 19 and 83880 DF,  p-value: < 2.2e-16
```

```
lmVets_inf_base=data.frame(age=30, weightKG=70, heightCM = 170, race="white", chol
era  = FALSE,cystitis  = FALSE,dengue  = FALSE,fever_unspec  = FALSE,gastro  = FAL
SE,goiter  = FALSE, hepatitis  = FALSE,influenza  = FALSE,malaria  = FALSE,nephrit
is  = FALSE,osteomyelitis  = FALSE,plague  = FALSE,pneumonia  = FALSE, rheumatic_f
ever  = FALSE,scarlet_fever  = FALSE,sepsis  = FALSE,smallpox   = FALSE,syphilis =
FALSE,tb = FALSE, typhoid = FALSE, typhus = FALSE)


disease_names <- c("cholera", "cystitis ", "dengue", "fever_unspec", "gastro", "go
iter", "hepatitis ", "influenza", "malaria", "nephritis", "pneumonia", "scarlet_fe
ver", "sepsis", "smallpox", "syphilis", "tb")

df <- NULL
for (dn in disease_names) {
  lmVets_inf_dn <- lmVets_inf_base
  lmVets_inf_dn[dn] <- TRUE
  pp <- round(predict(lmVets_inf, lmVets_inf_dn, interval="confidence"),2)
  row <- data.frame(disease=dn,fit=pp[1],lower=pp[2],upper=pp[3])
  if (is.null(df)) {
    df <- row
  } else {
    df <- rbind(df,row)
  }
}
write.csv (df,"~/Desktop/Temperature study/diseases.csv")
```

```
subset_no_inf_subset <- !dataVeterans$tb & !dataVeterans$syphilis & !dataVeterans$
osteomyelitis & !dataVeterans$pneumonia & !dataVeterans$goiter & !dataVeterans$gas
tro & !dataVeterans$nephritis   &  !dataVeterans$cystitis  & !dataVeterans$malaria
&!dataVeterans$cholera & !dataVeterans$cystitis & !dataVeterans$dengue & !dataVete
rans$fever_unspec  & !dataVeterans$hepatitis & !dataVeterans$influenza &!dataVeter
ans$malaria & !dataVeterans$plague &  !dataVeterans$rheumatic_fever & !dataVeteran
s$scarlet_fever & !dataVeterans$sepsis & !dataVeterans$smallpox  & !dataVeterans$t
yphoid & !dataVeterans$typhus

lmVets_noinf <- lm(temp_C ~ age + weightKG+ heightCM, data=dataVeterans[subset_no_
inf_subset,])

summary(lmVets_noinf)
```

```
##
## Call:
## lm(formula = temp_C ~ age + weightKG + heightCM, data = dataVeterans[subset_no_
inf_subset,
##     ])
##
## Residuals:
##      Min       1Q   Median       3Q      Max
## -1.82611 -0.07898 -0.00595  0.06760  2.06418
##
## Coefficients:
##               Estimate Std. Error  t value Pr(>|t|)
## (Intercept)  3.710e+01  2.489e-02 1490.479   <2e-16 ***
## age         -2.892e-03  1.047e-04  -27.606   <2e-16 ***
## weightKG    -1.061e-04  9.456e-05   -1.122    0.262
## heightCM     9.447e-05  1.460e-04    0.647    0.518
## ---
## Signif. codes:  0 '***' 0.001 '**' 0.01 '*' 0.05 '.' 0.1 ' ' 1
##
## Residual standard error: 0.257 on 77591 degrees of freedom
## Multiple R-squared:  0.009869,   Adjusted R-squared:  0.009831
## F-statistic: 257.8 on 3 and 77591 DF,  p-value: < 2.2e-16
```

```
round(predict(lmVets_noinf,lmVets_inf_base, interval="predict"),2)
```

```
##     fit   lwr   upr
## 1 37.02 36.52 37.53
```

```
subset_no_TB_subset <- !dataVeterans$tb & !dataVeterans$pneumonia


lmVets_noTB <- lm(temp_C ~ age + weightKG+ heightCM+race, data=dataVeterans[subset
_no_TB_subset,])
lmVets_everyone <- lm(temp_C ~ age + weightKG+ heightCM+race, data=dataVeterans[,]
)


summary(lmVets_noTB)
```

```
##
## Call:
## lm(formula = temp_C ~ age + weightKG + heightCM + race, data = dataVeterans[sub
set_no_TB_subset,
##     ])
##
## Residuals:
##      Min       1Q   Median       3Q      Max
## -1.83122 -0.08112 -0.00590  0.06886  2.06007
##
## Coefficients:
##               Estimate Std. Error  t value Pr(>|t|)
## (Intercept)  3.714e+01  2.440e-02 1521.680   <2e-16 ***
## age         -2.910e-03  1.020e-04  -28.535   <2e-16 ***
## weightKG    -2.976e-05  9.220e-05   -0.323    0.747
## heightCM    -8.963e-05  1.435e-04   -0.625    0.532
## raceblack   -2.178e-02  2.101e-03  -10.367   <2e-16 ***
## ---
## Signif. codes:  0 '***' 0.001 '**' 0.01 '*' 0.05 '.' 0.1 ' ' 1
##
## Residual standard error: 0.258 on 82902 degrees of freedom
## Multiple R-squared:  0.01143,    Adjusted R-squared:  0.01138
## F-statistic: 239.7 on 4 and 82902 DF,  p-value: < 2.2e-16
```

```
predict(lmVets_noTB,lmVets_inf_base)-predict(lmVets_everyone,lmVets_inf_base)
```

```
##            1
## -0.003154516
```

```
predict(lmVets_noTB,lmVets_inf_base)
```

```
##        1
## 37.03048
```

Analisys for black and white race subgroups separately. Figure 3S Table A and B.

```
lmVeterans_whites <-lm (temp_C~ age + weightKG + heightCM , data= dataVeterans [da
taVeterans$race == "white",])
summary(lmVeterans_whites)
```

```
##
## Call:
## lm(formula = temp_C ~ age + weightKG + heightCM, data = dataVeterans[dataVetera
ns$race ==
##     "white", ])
##
## Residuals:
##      Min       1Q   Median       3Q      Max
## -1.84366 -0.09321 -0.01258  0.07552  2.05850
##
## Coefficients:
##               Estimate Std. Error  t value Pr(>|t|)
## (Intercept)  3.717e+01  2.964e-02 1253.874   <2e-16 ***
## age         -3.534e-03  1.223e-04  -28.906   <2e-16 ***
## weightKG    -2.282e-04  1.092e-04   -2.089   0.0367 *
## heightCM    -3.412e-06  1.730e-04   -0.020   0.9843
## ---
## Signif. codes:  0 '***' 0.001 '**' 0.01 '*' 0.05 '.' 0.1 ' ' 1
##
## Residual standard error: 0.2738 on 63029 degrees of freedom
## Multiple R-squared:  0.01319,    Adjusted R-squared:  0.01314
## F-statistic: 280.8 on 3 and 63029 DF,  p-value: < 2.2e-16
```

```
lmVeterans_whites_predict =data.frame(age=30, weightKG=70, heightCM = 170, race="w
hite")

round(predict(lmVeterans_whites, lmVeterans_whites_predict, interval="confidence")
,2)
```

```
##     fit   lwr   upr
## 1 37.05 37.04 37.06
```

```
lmVeterans_blacks <-lm (temp_C~ age + weightKG + heightCM , data= dataVeterans [da
taVeterans$race == "black",])
summary(lmVeterans_blacks)
```

```
##
## Call:
## lm(formula = temp_C ~ age + weightKG + heightCM, data = dataVeterans[dataVetera
ns$race ==
##     "black", ])
##
## Residuals:
##      Min       1Q   Median       3Q      Max
## -1.58388 -0.04803  0.01149  0.06599  1.97484
##
## Coefficients:
##               Estimate Std. Error t value Pr(>|t|)
## (Intercept)  3.700e+01  4.121e-02 897.758  < 2e-16 ***
## age         -1.105e-03  1.819e-04  -6.079 1.23e-09 ***
## weightKG    -1.369e-04  1.713e-04  -0.799    0.424
## heightCM     2.083e-05  2.508e-04   0.083    0.934
## ---
## Signif. codes:  0 '***' 0.001 '**' 0.01 '*' 0.05 '.' 0.1 ' ' 1
##
## Residual standard error: 0.2198 on 20863 degrees of freedom
## Multiple R-squared:  0.001781,   Adjusted R-squared:  0.001638
## F-statistic: 12.41 on 3 and 20863 DF,  p-value: 4.175e-08
```

```
lmVeterans_b_predict =data.frame(age=30, weightKG=70, heightCM = 170, race="black"
)
round(predict(lmVeterans_blacks, lmVeterans_b_predict, interval="confidence"),2)
```

```
##     fit   lwr   upr
## 1 36.96 36.95 36.97
```

```
lmNHANES_W_HR <- lm(temp_C ~ age +  weight_KG + height_CM + time_HR, data=dataNHAN
ES[dataNHANES$sex == "male" & dataNHANES$race == "white", ], weights=dataNHANES$sa
mple_weights[dataNHANES$sex=="male" & dataNHANES$race == "white"])
summary(lmNHANES_W_HR)
```

```
##
## Call:
## lm(formula = temp_C ~ age + weight_KG + height_CM + time_HR,
##     data = dataNHANES[dataNHANES$sex == "male" & dataNHANES$race ==
##         "white", ], weights = dataNHANES$sample_weights[dataNHANES$sex ==
##         "male" & dataNHANES$race == "white"])
##
## Weighted Residuals:
##      Min       1Q   Median       3Q      Max
## -164.257    0.000    0.000    7.267  142.980
##
## Coefficients:
##               Estimate Std. Error t value Pr(>|t|)
## (Intercept) 36.8264755  0.1953783 188.488  < 2e-16 ***
## age         -0.0028352  0.0005801  -4.888 1.13e-06 ***
## weight_KG    0.0020464  0.0005891   3.473 0.000529 ***
## height_CM   -0.0012627  0.0011446  -1.103 0.270133
## time_HR      0.0129788  0.0017657   7.350 3.26e-13 ***
## ---
## Signif. codes:  0 '***' 0.001 '**' 0.01 '*' 0.05 '.' 0.1 ' ' 1
##
## Residual standard error: 31.65 on 1471 degrees of freedom
##   (2458 observations deleted due to missingness)
## Multiple R-squared:  0.06403,    Adjusted R-squared:  0.06148
## F-statistic: 25.16 on 4 and 1471 DF,  p-value: < 2.2e-16
```

```
nobs(lmNHANES_W_HR)
```

```
## [1] 1476
```

```
NHANES_predict=data.frame (age=30, weight_KG=70, height_CM = 170, time_HR=12, race
="white", sex ="male")
round(predict(lmNHANES_W_HR, NHANES_predict, interval="confidence"),2)
```

```
##     fit  lwr   upr
## 1 36.83 36.8 36.86
```

```
lmNHANES_W_black_HR <- lm(temp_C ~ age + weight_KG + height_CM + time_HR, data=dat
aNHANES[dataNHANES$sex == "male" & dataNHANES$race == "black", ], weights=dataNHAN
ES$sample_weights[dataNHANES$sex=="male" & dataNHANES$race == "black"])
summary(lmNHANES_W_black_HR)
```

```
##
## Call:
## lm(formula = temp_C ~ age + weight_KG + height_CM + time_HR,
##     data = dataNHANES[dataNHANES$sex == "male" & dataNHANES$race ==
##         "black", ], weights = dataNHANES$sample_weights[dataNHANES$sex ==
##         "male" & dataNHANES$race == "black"])
##
## Weighted Residuals:
##      Min       1Q   Median       3Q      Max
## -140.738   -5.530    0.000    6.196  140.373
##
## Coefficients:
##              Estimate Std. Error t value Pr(>|t|)
## (Intercept) 36.340576   0.526816  68.982  < 2e-16 ***
## age         -0.001111   0.001695  -0.656  0.51269
## weight_KG   -0.003098   0.001051  -2.949  0.00348 **
## height_CM    0.003242   0.003115   1.041  0.29889
## time_HR      0.015248   0.005196   2.935  0.00363 **
## ---
## Signif. codes:  0 '***' 0.001 '**' 0.01 '*' 0.05 '.' 0.1 ' ' 1
##
## Residual standard error: 27.96 on 265 degrees of freedom
##   (494 observations deleted due to missingness)
## Multiple R-squared:  0.04992,    Adjusted R-squared:  0.03558
## F-statistic: 3.481 on 4 and 265 DF,  p-value: 0.008607
```

```
nobs(lmNHANES_W_black_HR)
```

```
## [1] 270
```

```
NHANES_predict_black=data.frame (age=30, weight_KG=70, height_CM = 170, race="blac
k", sex ="male", time_HR= 12)
round(predict(lmNHANES_W_black_HR, NHANES_predict_black, interval="confidence"),2)
```

```
##     fit   lwr  upr
## 1 36.82 36.75 36.9
```

```
lmNHANES_W_F_HR <- lm(temp_C ~ age +  weight_KG + height_CM + time_HR, data=dataNH
ANES[dataNHANES$sex == "female" & dataNHANES$race == "white", ], weights=dataNHANE
S$sample_weights[dataNHANES$sex=="female" & dataNHANES$race == "white"])
summary(lmNHANES_W_F_HR)
```

```
##
## Call:
## lm(formula = temp_C ~ age + weight_KG + height_CM + time_HR,
##     data = dataNHANES[dataNHANES$sex == "female" & dataNHANES$race ==
##         "white", ], weights = dataNHANES$sample_weights[dataNHANES$sex ==
##         "female" & dataNHANES$race == "white"])
##
## Weighted Residuals:
##      Min       1Q   Median       3Q      Max
## -172.796    0.000    0.000    5.568  133.029
##
## Coefficients:
##               Estimate Std. Error t value Pr(>|t|)
## (Intercept)  3.690e+01  2.016e-01 183.035  < 2e-16 ***
## age         -3.294e-03  6.080e-04  -5.418 6.93e-08 ***
## weight_KG    9.552e-05  5.206e-04   0.183    0.854
## height_CM   -4.691e-04  1.213e-03  -0.387    0.699
## time_HR      1.090e-02  1.924e-03   5.664 1.75e-08 ***
## ---
## Signif. codes:  0 '***' 0.001 '**' 0.01 '*' 0.05 '.' 0.1 ' ' 1
##
## Residual standard error: 28.23 on 1608 degrees of freedom
##   (4616 observations deleted due to missingness)
## Multiple R-squared:  0.03837,    Adjusted R-squared:  0.03597
## F-statistic: 16.04 on 4 and 1608 DF,  p-value: 6.96e-13
```

```
nobs(lmNHANES_W_F_HR)
```

```
## [1] 1613
```

```
NHANES_predict=data.frame (age=30, weight_KG=70, height_CM = 170, time_HR=12, race
="white", sex ="female")
round(predict(lmNHANES_W_F_HR , NHANES_predict, interval="confidence"),2)
```

```
##     fit   lwr   upr
## 1 36.85 36.82 36.89
```

```
lmNHANES_W_F_HR_black <- lm(temp_C ~ age +  weight_KG + height_CM + time_HR, data=
dataNHANES[dataNHANES$sex == "female" & dataNHANES$race == "black", ], weights=dat
aNHANES$sample_weights[dataNHANES$sex=="female" & dataNHANES$race == "black"])
summary(lmNHANES_W_F_HR_black)
```

```
##
## Call:
## lm(formula = temp_C ~ age + weight_KG + height_CM + time_HR,
##     data = dataNHANES[dataNHANES$sex == "female" & dataNHANES$race ==
##         "black", ], weights = dataNHANES$sample_weights[dataNHANES$sex ==
##         "female" & dataNHANES$race == "black"])
##
## Weighted Residuals:
##      Min       1Q   Median       3Q      Max
## -201.600   -2.218    0.000    7.178   68.604
##
## Coefficients:
##               Estimate Std. Error t value Pr(>|t|)
## (Intercept) 36.1756297  0.4689283  77.145  < 2e-16 ***
## age         -0.0018158  0.0014596  -1.244  0.21440
## weight_KG    0.0020636  0.0007774   2.655  0.00834 **
## height_CM    0.0038749  0.0028296   1.369  0.17184
## time_HR     -0.0025030  0.0047541  -0.526  0.59892
## ---
## Signif. codes:  0 '***' 0.001 '**' 0.01 '*' 0.05 '.' 0.1 ' ' 1
##
## Residual standard error: 21.91 on 316 degrees of freedom
##   (1082 observations deleted due to missingness)
## Multiple R-squared:  0.03965,    Adjusted R-squared:  0.0275
## F-statistic: 3.262 on 4 and 316 DF,  p-value: 0.01216
```

```
nobs(lmNHANES_W_F_HR_black)
```

```
## [1] 321
```

```
NHANES_predict_b_f=data.frame (age=30, weight_KG=70, height_CM = 170, time_HR=12,
race="white", sex ="female")
round(predict(lmNHANES_W_F_HR_black , NHANES_predict_b_f, interval="confidence"),2
)
```

```
##     fit   lwr   upr
## 1 36.89 36.82 36.97
```

```
 lmSTRIDE_HR <- lm(temp_C ~ age_years +  weight_KG + height_CM + time_HR, data=ST_
data[ST_data$GENDER == "Male" & ST_data$race == "White", ])
summary(lmSTRIDE_HR)
```

```
##
## Call:
## lm(formula = temp_C ~ age_years + weight_KG + height_CM + time_HR,
##     data = ST_data[ST_data$GENDER == "Male" & ST_data$race ==
##         "White", ])
##
## Residuals:
##      Min       1Q   Median       3Q      Max
## -1.69423 -0.20102  0.00887  0.21052  2.42194
##
## Coefficients:
##               Estimate Std. Error t value Pr(>|t|)
## (Intercept)  3.674e+01  2.617e-02 1404.04   <2e-16 ***
## age_years   -2.998e-03  6.802e-05  -44.08   <2e-16 ***
## weight_KG    1.255e-03  6.197e-05   20.25   <2e-16 ***
## height_CM   -1.868e-03  1.481e-04  -12.62   <2e-16 ***
## time_HR      2.164e-02  3.666e-04   59.01   <2e-16 ***
## ---
## Signif. codes:  0 '***' 0.001 '**' 0.01 '*' 0.05 '.' 0.1 ' ' 1
##
## Residual standard error: 0.3595 on 127108 degrees of freedom
##   (19414 observations deleted due to missingness)
## Multiple R-squared:  0.04446,    Adjusted R-squared:  0.04443
## F-statistic:  1479 on 4 and 127108 DF,  p-value: < 2.2e-16
```

```
nobs(lmSTRIDE_HR)
```

```
## [1] 127113
```

```
STRIDE_predict=data.frame(age_years=30, weight_KG=70, height_CM = 170, time_HR=12,
race="White", GENDER = "Male")

round(predict(lmSTRIDE_HR, STRIDE_predict, interval="confidence"),2)
```

```
##     fit   lwr   upr
## 1 36.68 36.68 36.69
```

```
 lmSTRIDE_HR_B <- lm(temp_C ~ age_years +  weight_KG + height_CM + time_HR, data=S
T_data[ST_data$GENDER == "Male" & ST_data$race == "Black", ])
summary(lmSTRIDE_HR_B)
```

```
##
## Call:
## lm(formula = temp_C ~ age_years + weight_KG + height_CM + time_HR,
##     data = ST_data[ST_data$GENDER == "Male" & ST_data$race ==
##         "Black", ])
##
## Residuals:
##      Min       1Q   Median       3Q      Max
## -1.57136 -0.23133  0.02297  0.24755  2.45406
##
## Coefficients:
##               Estimate Std. Error t value Pr(>|t|)
## (Intercept) 36.5236062  0.0753251 484.880  < 2e-16 ***
## age_years   -0.0029215  0.0002071 -14.109  < 2e-16 ***
## weight_KG    0.0008483  0.0001608   5.275 1.35e-07 ***
## height_CM   -0.0010565  0.0004380  -2.412   0.0159 *
## time_HR      0.0256084  0.0011256  22.751  < 2e-16 ***
## ---
## Signif. codes:  0 '***' 0.001 '**' 0.01 '*' 0.05 '.' 0.1 ' ' 1
##
## Residual standard error: 0.3789 on 15395 degrees of freedom
##   (19414 observations deleted due to missingness)
## Multiple R-squared:  0.04982,    Adjusted R-squared:  0.04957
## F-statistic: 201.8 on 4 and 15395 DF,  p-value: < 2.2e-16
```

```
nobs(lmSTRIDE_HR_B)
```

```
## [1] 15400
```

```
STRIDE_predict_B=data.frame(age_years=30, weight_KG=70, height_CM = 170, time_HR=1
2, race="Black", GENDER = "Male")

round(predict(lmSTRIDE_HR_B, STRIDE_predict_B, interval="confidence"),2)
```

```
##     fit   lwr   upr
## 1 36.62 36.61 36.64
```

```
 lmSTRIDE_HR_B_F <- lm(temp_C ~ age_years +  weight_KG + height_CM + time_HR, data
=ST_data[ST_data$GENDER == "Female" & ST_data$race == "Black", ])
summary(lmSTRIDE_HR_B_F)
```

```
##
## Call:
## lm(formula = temp_C ~ age_years + weight_KG + height_CM + time_HR,
##     data = ST_data[ST_data$GENDER == "Female" & ST_data$race ==
##         "Black", ])
##
## Residuals:
##      Min       1Q   Median       3Q      Max
## -1.62550 -0.21009  0.02439  0.23212  2.41760
##
## Coefficients:
##               Estimate Std. Error t value Pr(>|t|)
## (Intercept) 36.8608501  0.0516321 713.914  < 2e-16 ***
## age_years   -0.0045661  0.0001369 -33.349  < 2e-16 ***
## weight_KG    0.0004086  0.0001125   3.633  0.00028 ***
## height_CM   -0.0017117  0.0003125  -5.478 4.34e-08 ***
## time_HR      0.0233365  0.0007957  29.328  < 2e-16 ***
## ---
## Signif. codes:  0 '***' 0.001 '**' 0.01 '*' 0.05 '.' 0.1 ' ' 1
##
## Residual standard error: 0.3688 on 30284 degrees of freedom
##   (25584 observations deleted due to missingness)
## Multiple R-squared:  0.06423,    Adjusted R-squared:  0.06411
## F-statistic: 519.7 on 4 and 30284 DF,  p-value: < 2.2e-16
```

```
nobs(lmSTRIDE_HR_B_F)
```

```
## [1] 30289
```

```
STRIDE_predict_B_F=data.frame(age_years=30, weight_KG=70, height_CM = 170, time_HR
=12, race="Black", GENDER = "Female")

round(predict(lmSTRIDE_HR_B_F, STRIDE_predict_B_F, interval="confidence"),2)
```

```
##     fit   lwr   upr
## 1 36.74 36.73 36.75
```

```
 lmSTRIDE_F_HR <- lm(temp_C ~ age_years + weight_KG + height_CM + time_HR ,data=ST
_data[ST_data$GENDER == "Female" & ST_data$race == "White", ])
summary(lmSTRIDE_F_HR)
```

```
##
## Call:
## lm(formula = temp_C ~ age_years + weight_KG + height_CM + time_HR,
##     data = ST_data[ST_data$GENDER == "Female" & ST_data$race ==
##         "White", ])
##
## Residuals:
##      Min       1Q   Median       3Q      Max
## -1.70509 -0.20319 -0.00088  0.20525  2.37776
##
## Coefficients:
##               Estimate Std. Error t value Pr(>|t|)
## (Intercept)  3.677e+01  2.104e-02 1747.93   <2e-16 ***
## age_years   -3.640e-03  5.494e-05  -66.25   <2e-16 ***
## weight_KG    8.071e-04  4.991e-05   16.17   <2e-16 ***
## height_CM   -1.129e-03  1.249e-04   -9.04   <2e-16 ***
## time_HR      2.014e-02  3.163e-04   63.67   <2e-16 ***
## ---
## Signif. codes:  0 '***' 0.001 '**' 0.01 '*' 0.05 '.' 0.1 ' ' 1
##
## Residual standard error: 0.3552 on 178463 degrees of freedom
##   (25584 observations deleted due to missingness)
## Multiple R-squared:  0.04716,    Adjusted R-squared:  0.04714
## F-statistic:  2208 on 4 and 178463 DF,  p-value: < 2.2e-16
```

```
STRIDE_predict=data.frame(age_years=30, weight_KG=70, height_CM = 170, time_HR=12,
race="White", GENDER = "Female")

round(predict(lmSTRIDE_F_HR, STRIDE_predict, interval="confidence"),2)
```

```
##     fit   lwr   upr
## 1 36.77 36.77 36.77
```

Analysis using BMI adjusted for height. Table S2

```
lm_bmi_adj <- lm(BMI ~ heightCM, data= dataVeterans)
dataVeterans$bmi_adj <- residuals(lm_bmi_adj)
summary (lm_bmi_adj)
```

```
##
## Call:
## lm(formula = BMI ~ heightCM, data = dataVeterans)
##
## Residuals:
##      Min       1Q   Median       3Q      Max
## -12.8317  -2.1699  -0.5072   1.5506  31.3934
##
## Coefficients:
##             Estimate Std. Error t value Pr(>|t|)
## (Intercept) 36.47411    0.28625  127.42   <2e-16 ***
## heightCM    -0.07754    0.00166  -46.72   <2e-16 ***
## ---
## Signif. codes:  0 '***' 0.001 '**' 0.01 '*' 0.05 '.' 0.1 ' ' 1
##
## Residual standard error: 3.29 on 83898 degrees of freedom
## Multiple R-squared:  0.02536,    Adjusted R-squared:  0.02535
## F-statistic:  2183 on 1 and 83898 DF,  p-value: < 2.2e-16
```

```
base_h <- 170
base_w <- 70
base_bmi <- base_w / (base_h/100)^2
base_bmi_adj <- base_bmi - (predict(lm_bmi_adj,data.frame(heightCM=base_h)))
```

```
lmVets_bmi_adj <-lm(temp_C ~ age + heightCM  + bmi_adj+ race, data=dataVeterans )
summary(lmVets_bmi_adj)
```

```
##
## Call:
## lm(formula = temp_C ~ age + heightCM + bmi_adj + race, data = dataVeterans)
##
## Residuals:
##      Min       1Q   Median       3Q      Max
## -1.83508 -0.08300 -0.00716  0.06856  2.05630
##
## Coefficients:
##               Estimate Std. Error  t value Pr(>|t|)
## (Intercept) 37.1429591  0.0243991 1522.310   <2e-16 ***
## age         -0.0029873  0.0001028  -29.065   <2e-16 ***
## heightCM    -0.0001123  0.0001336   -0.840   0.4007
## bmi_adj     -0.0006561  0.0002757   -2.380   0.0173 *
## raceblack   -0.0209699  0.0021148   -9.916   <2e-16 ***
## ---
## Signif. codes:  0 '***' 0.001 '**' 0.01 '*' 0.05 '.' 0.1 ' ' 1
##
## Residual standard error: 0.2616 on 83895 degrees of freedom
## Multiple R-squared:  0.0116, Adjusted R-squared:  0.01156
## F-statistic: 246.2 on 4 and 83895 DF,  p-value: < 2.2e-16
```

```
lmVeterans_predict=data.frame(age=30, weightKG=70, heightCM = 170, bmi_adj=base_bm
i_adj, race="white")

round(predict(lmVets_bmi_adj, lmVeterans_predict, interval="confidence"),2)
```

```
##     fit   lwr   upr
## 1 37.03 37.03 37.04
```

```
dataNHANES$bmi_adj <- residuals(lm(BMI ~ height_CM, data=dataNHANES))

lmNHANES_BMI_adj_F <-lm(temp_C ~ age + height_CM  + bmi_adj + race + time_HR, data
=dataNHANES[dataNHANES$sex == "female", ],weights=dataNHANES$sample_weights[dataNH
ANES$sex=="female" ])
summary(lmNHANES_BMI_adj_F)
```

```
##
## Call:
## lm(formula = temp_C ~ age + height_CM + bmi_adj + race + time_HR,
##     data = dataNHANES[dataNHANES$sex == "female", ], weights = dataNHANES$sampl
e_weights[dataNHANES$sex ==
##         "female"])
##
## Weighted Residuals:
##      Min       1Q   Median       3Q      Max
## -215.191    0.000    0.000    6.133  133.348
##
## Coefficients:
##               Estimate Std. Error t value Pr(>|t|)
## (Intercept) 36.7656077  0.1834657 200.395  < 2e-16 ***
## age         -0.0030663  0.0005588  -5.487 4.63e-08 ***
## height_CM    0.0003931  0.0010721   0.367    0.714
## bmi_adj      0.0013067  0.0011571   1.129    0.259
## raceblack    0.0106378  0.0228308   0.466    0.641
## raceother    0.0546304  0.0748077   0.730    0.465
## time_HR      0.0099372  0.0017707   5.612 2.29e-08 ***
## ---
## Signif. codes:  0 '***' 0.001 '**' 0.01 '*' 0.05 '.' 0.1 ' ' 1
##
## Residual standard error: 27.34 on 1940 degrees of freedom
##   (5774 observations deleted due to missingness)
## Multiple R-squared:  0.0331, Adjusted R-squared:  0.03011
## F-statistic: 11.07 on 6 and 1940 DF,  p-value: 3.633e-12
```

```
NHANES_predict=data.frame (age=30, weight_KG=70, height_CM = 170, bmi_adj=base_bmi
_adj, race="white", sex ="female", time_HR=12)
round(predict(lmNHANES_BMI_adj_F, NHANES_predict, interval="confidence"),2)
```

```
##     fit   lwr   upr
## 1 36.86 36.83 36.89
```

```
lmNHANES_BMI_adj <-lm(temp_C ~ age + height_CM  + bmi_adj + race +time_HR, data=da
taNHANES[dataNHANES$sex == "male", ],weights=dataNHANES$sample_weights[dataNHANES$
sex=="male" ])
summary(lmNHANES_BMI_adj)
```

```
##
## Call:
## lm(formula = temp_C ~ age + height_CM + bmi_adj + race + time_HR,
##     data = dataNHANES[dataNHANES$sex == "male", ], weights = dataNHANES$sample_
weights[dataNHANES$sex ==
##         "male"])
##
## Weighted Residuals:
##      Min       1Q   Median       3Q      Max
## -181.330   -0.167    0.000    7.164  143.992
##
## Coefficients:
##               Estimate Std. Error t value Pr(>|t|)
## (Intercept) 36.6806505  0.1779711 206.105  < 2e-16 ***
## age         -0.0025628  0.0005414  -4.733 2.38e-06 ***
## height_CM    0.0004558  0.0009690   0.470   0.6382
## bmi_adj      0.0029306  0.0015667   1.871   0.0616 .
## raceblack   -0.0101492  0.0234593  -0.433   0.6653
## raceother    0.1191273  0.0663340   1.796   0.0727 .
## time_HR      0.0124758  0.0016483   7.569 6.04e-14 ***
## ---
## Signif. codes:  0 '***' 0.001 '**' 0.01 '*' 0.05 '.' 0.1 ' ' 1
##
## Residual standard error: 31.15 on 1759 degrees of freedom
##   (2995 observations deleted due to missingness)
## Multiple R-squared:  0.05317,    Adjusted R-squared:  0.04994
## F-statistic: 16.46 on 6 and 1759 DF,  p-value: < 2.2e-16
```

```
NHANES_predict=data.frame (age=30, weight_KG=70, height_CM = 170, bmi_adj=base_bmi
_adj, race="white", sex ="male", time_HR=12)
round(predict(lmNHANES_BMI_adj, NHANES_predict, interval="confidence"),2)
```

```
##     fit   lwr   upr
## 1 36.83 36.81 36.86
```

```
ST_data$bmi_adj <- residuals(lm(BMI ~ height_CM, data=ST_data))
lmSTRIDE_BMI_adj_F <-lm(temp_C ~ age_years + height_CM  + bmi_adj + race +time_HR,
data=ST_data[ST_data$GENDER  == "Female" , ])

summary (lmSTRIDE_BMI_adj_F)
```

```
##
## Call:
## lm(formula = temp_C ~ age_years + height_CM + bmi_adj + race +
##     time_HR, data = ST_data[ST_data$GENDER == "Female", ])
##
## Residuals:
##      Min       1Q   Median       3Q      Max
## -1.73641 -0.20149  0.00159  0.20628  2.40134
##
## Coefficients:
##               Estimate Std. Error  t value Pr(>|t|)
## (Intercept)  3.673e+01  1.603e-02 2291.522  < 2e-16 ***
## age_years   -3.675e-03  4.119e-05  -89.229  < 2e-16 ***
## height_CM   -5.333e-04  9.213e-05   -5.789 7.10e-09 ***
## bmi_adj      1.869e-03  1.013e-04   18.446  < 2e-16 ***
## raceBlack   -5.214e-02  2.249e-03  -23.182  < 2e-16 ***
## raceOther   -7.291e-03  1.460e-03   -4.995 5.88e-07 ***
## time_HR      2.106e-02  2.346e-04   89.771  < 2e-16 ***
## ---
## Signif. codes:  0 '***' 0.001 '**' 0.01 '*' 0.05 '.' 0.1 ' ' 1
##
## Residual standard error: 0.3564 on 322415 degrees of freedom
##   (25584 observations deleted due to missingness)
## Multiple R-squared:  0.05083,    Adjusted R-squared:  0.05082
## F-statistic:  2878 on 6 and 322415 DF,  p-value: < 2.2e-16
```

```
STRIDE_predict=data.frame(age_years=30, weight_KG=70, height_CM = 170, time_HR=12,
bmi_adj=base_bmi_adj, race="White", GENDER = "Female")

round(predict(lmSTRIDE_BMI_adj_F, STRIDE_predict, interval="confidence"),2)
```

```
##     fit   lwr   upr
## 1 36.78 36.78 36.78
```

```
lmSTRIDE_BMI_adj <-lm(temp_C ~ age_years + height_CM  + bmi_adj + race + time_HR,
data=ST_data[ST_data$GENDER  == "Male" , ])

summary (lmSTRIDE_BMI_adj)
```

```
##
## Call:
## lm(formula = temp_C ~ age_years + height_CM + bmi_adj + race +
##     time_HR, data = ST_data[ST_data$GENDER == "Male", ])
##
## Residuals:
##      Min       1Q   Median       3Q      Max
## -1.70519 -0.20291  0.00938  0.21150  2.45284
##
## Coefficients:
##               Estimate Std. Error  t value Pr(>|t|)
## (Intercept)  3.655e+01  2.028e-02 1802.072  < 2e-16 ***
## age_years   -2.932e-03  5.312e-05  -55.198  < 2e-16 ***
## height_CM   -2.954e-04  1.075e-04   -2.748  0.00599 **
## bmi_adj      3.572e-03  1.548e-04   23.073  < 2e-16 ***
## raceBlack   -6.015e-02  3.107e-03  -19.361  < 2e-16 ***
## raceOther   -5.460e-04  1.886e-03   -0.289  0.77221
## time_HR      2.310e-02  2.874e-04   80.367  < 2e-16 ***
## ---
## Signif. codes:  0 '***' 0.001 '**' 0.01 '*' 0.05 '.' 0.1 ' ' 1
##
## Residual standard error: 0.3634 on 210795 degrees of freedom
##   (19414 observations deleted due to missingness)
## Multiple R-squared:  0.0492, Adjusted R-squared:  0.04917
## F-statistic:  1818 on 6 and 210795 DF,  p-value: < 2.2e-16
```

```
STRIDE_predict=data.frame(age_years=30, weight_KG=70, height_CM = 170, time_HR=12,
bmi_adj=base_bmi_adj, race="White", GENDER = "Male")
round(predict(lmSTRIDE_BMI_adj, STRIDE_predict, interval="confidence"),2)
```

```
##     fit   lwr  upr
## 1 36.69 36.69 36.7
```
