# Supplementary material for: Decreasing human body temperature in the United States since the Industrial Revolution
Source: eLife. 2020 Jan 7;9:e49555. doi: 10.7554/eLife.49555 (PMC6946399; doi:10.7554/eLife.49555)
Supplement: Supplementary file 1. — Expected body temperature (and associated 95% confidence interval) for a 30 year old white man with body weight of 70 kg and height 170 cm in the UAVCW cohort. *, **, *** indicates significance at the 90%, 95%, and 99% level, respectively. [file elife-49555-supp1.docx]

| **Infectious condition** | **Timing** | **N** | **Predicted temperature, ^o^C (95% CI)** |
| --- | --- | --- | --- |
| None |  | 77,595 | 37.02 (36.52 - 37.53) |
| Cholera | Ever in medical history | 8 | 36.99 (36.81 - 37.17) |
| Cystitis | Current | 1,301 | 37.03 (37.02 - 37.03) |
| Dengue | Ever in medical history | 35 | 36.95 (36.87 - 37.04) |
| Fever (unspecified) | Ever in medical history | 24 | 36.76 (36.66 - 36.87)*** |
| Gastro-intestinal infection | Current | 938 | 37.04 (37.02 - 37.06) |
| Goiter | Current | 69 | 37.02 (36.96 - 37.08) |
| Hepatitis | Ever in medical history | 24 | 37.03 (37.02 - 37.03) |
| Influenza | Ever in medical history | 69 | 37.06 (37 - 37.12) |
| Malaria | Ever in medical history | 2,023 | 37.03 (37.02 - 37.05) |
| Nephritis | Current | 201 | 37.03 (37.00 - 37.07) |
| Pneumonia | Current | 277 | 37.06 (37.03 - 37.09) * |
| Scarlet fever | Ever in medical history | 10 | 37.02 (36.85 - 37.18) |
| Sepsis | Ever in medical history | 53 | 37.04 (36.97 - 37.11) |
| Smallpox | Ever in medical history | 385 | 37.03 (37.01 - 37.06) |
| Syphilis | Ever in medical history | 465 | 37.05 (37.02 - 37.07) |
| Tuberculosis | Current | 738 | 37.22 (37.20 - 37.24 ) *** |
