# Supplementary material for: Decreasing human body temperature in the United States since the Industrial Revolution
Source: eLife. 2020 Jan 7;9:e49555. doi: 10.7554/eLife.49555 (PMC6946399; doi:10.7554/eLife.49555)
Supplement: Transparent reporting form [file elife-49555-transrepform.pdf]

eLIFE
1st Floor
24 Hills Road
Cambridge CB2 1JP, UK
P 01223 855340
W elifesciences.org
T @elife

# *eLife's* transparent reporting form

We encourage authors to provide detailed information *within their submission* to facilitate the interpretation and replication of experiments. Authors can upload supporting documentation to indicate the use of appropriate reporting guidelines for health-related research (see EQUATOR Network), life science research (see the BioSharing Information Resource), or the ARRIVE guidelines for reporting work involving animal research. Where applicable, authors should refer to any relevant reporting standards documents in this form.

If you have any questions, please consult our Journal Policies and/or contact us: editorial@elifesciences.org.

**Sample-size estimation**

- You should state whether an appropriate sample size was computed when the study was being designed
- You should state the statistical method of sample size computation and any required assumptions
- If no explicit power analysis was used, you should describe how you decided what sample (replicate) size (number) to use

Please outline where this information can be found within the submission (e.g., sections or figure legends), or explain why this information doesn't apply to your submission:

No sample size calculations were performed for this study as we elected to use all eligible observations within historical (locked) datasets from the years 1860-1940, 1971-1975 and 2007-2017. The cohorts followed in these time periods are described under MATERIALS AND METHODS, lines 193-229. Observations of fever, improbable values of temperature, weight and height, and those men unlikely to have been veterans of the Civil War were excluded from the analysis sample, as described in lines 230-236. Our final sample size included 677, 423 observations from 189,338 individuals spanning 155 years of measurement and 197 birth years (these totals and breakdown by cohort are presented in Table 1 (line 482)). Confidence intervals and standard errors were calculated from multivariate linear regression (Figures 1-3 and Figure 1-figure supplements 1-6 (lines 408-480)) and suggest a sufficiently large sample for precise estimates for most subgroups of interest within each cohort.

**Replicates**

- You should report how often each experiment was performed
- You should include a definition of biological versus technical replication
- The data obtained should be provided and sufficient information should be provided to indicate the number of independent biological and/or technical replicates
- If you encountered any outliers, you should describe how these were handled
- Criteria for exclusion/inclusion of data should be clearly stated
- High-throughput sequence data should be uploaded before submission, with a private link for reviewers provided (these are available from both GEO and ArrayExpress)

Please outline where this information can be found within the submission (e.g., sections or figure legends), or explain why this information doesn't apply to your submission:

eLife Sciences Publications, Ltd is a limited liability non-profit non-stock corporation incorporated in the State of Delaware, USA, with company number 5030732, and is registered in the UK with company number FC030576 and branch number BR015634 at the address 1st Floor, 24 Hills Road, Cambridge CB2 1JP | August 2014

Our study is an analysis of historical data and as such was not experimental, and did not include either biological or technical replicates. Participants in the UAVCW and STRIDE could have provided between 1 and 4 temperature measurements, but these were assessed at different time points/visits so are not replicates (Table 1). Inclusion criteria for each cohort can be found in the section MATERIALS AND METHODS, lines 193-229. Exclusion criteria and our handling of outliers can be found in the same section, lines 230-236. No high-throughput sequence data was involved in this analysis.

**Statistical reporting**

- Statistical analysis methods should be described and justified
- Raw data should be presented in figures whenever informative to do so (typically when N per group is less than 10)
- For each experiment, you should identify the statistical tests used, exact values of N, definitions of center, methods of multiple test correction, and dispersion and precision measures (e.g., mean, median, SD, SEM, confidence intervals; and, for the major substantive results, a measure of effect size (e.g., Pearson's r, Cohen's d)
- Report exact p-values wherever possible alongside the summary statistics and 95% confidence intervals. These should be reported for all key questions and not only when the p-value is less than 0.05.

Please outline where this information can be found within the submission (e.g., sections or figure legends), or explain why this information doesn’t apply to your submission:

Data and statistical analysis methods are described in detail under MATERIALS AND METHODS, lines 241-281. We present each proposed analysis, the method used to evaluate this analysis, and both the dependent and independent variables considered for each analysis. Table 1 (line 481) provides the total N and percent of total for each primary variable of interest (age, weight, height, sex and race, overall and for each cohort); the distribution of continuous variables is provided by cohort (mean, standard deviation). We have additionally provided a histogram of temperature in each cohort (Figure 1-figure supplement 1). Figures 1-3 (lines 407-437) present both coefficients and standard errors from multivariate linear regression, and statistical significance at 90% and 99% is indicated by * and ** respectively (no p-values are provided). Confidence levels of 95% are presented in each graph as a shaded area around each trend line. The Figure 1-supplements (lines 440-480) and Supplemental File 1 (lines 486- 490) provide further subset analyses, with both confidence intervals around point estimates and * marking levels of significance (90%, 95% and 99%).

(For large datasets, or papers with a very large number of statistical tests, you may upload a single table file with tests, Ns, etc., with reference to sections in the manuscript.)

**Group allocation**

- Indicate how samples were allocated into experimental groups (in the case of clinical studies, please specify allocation to treatment method); if randomization was used, please also state if restricted randomization was applied
- Indicate if masking was used during group allocation, data collection and/or data analysis

eLife Sciences Publications, Ltd is a limited liability non-profit non-stock corporation incorporated in the State of Delaware, USA, with company number 5030732, and is registered in the UK with company number FC030576 and branch number BR015634 at the address 1st Floor, 24 Hills Road, Cambridge CB2 1JP | August 2014

Please outline where this information can be found within the submission (e.g., sections or figure legends), or explain why this information doesn't apply to your submission:

Our study did not include experimental groups, and neither randomization nor masking were performed. Our analyses were limited to available historical demographic and clinical data, with variability in the information collected by cohort.

**Additional data files ("source data")**

- We encourage you to upload relevant additional data files, such as numerical data that are represented as a graph in a figure, or as a summary table
- Where provided, these should be in the most useful format, and they can be uploaded as "Source data" files linked to a main figure or table
- Include model definition files including the full list of parameters used
- Include code used for data analysis (e.g., R, MatLab)
- Avoid stating that data files are "available upon request"

Please indicate the figures or tables for which source data files have been provided:

We have included as additional files the specific cohort datasets and the R code used for all reported analyses. We include separately the code and data required for figures and tables both in the main text (Figures 1-3 and Table 1) and for the supplemental material (Figure 1-figure supplements 1-6 and Supplementary File 1).

eLife Sciences Publications, Ltd is a limited liability non-profit non-stock corporation incorporated in the State of Delaware, USA, with company number 5030732, and is registered in the UK with company number FC030576 and branch number BR015634 at the address 1st Floor, 24 Hills Road, Cambridge CB2 1JP | August 2014
